# Active GSK3β and an intact β-catenin TCF complex are essential for the differentiation of human myogenic progenitor cells

**DOI:** 10.1038/s41598-017-10731-1

**Published:** 2017-10-13

**Authors:** C. C. Agley, F. C. Lewis, O. Jaka, N. R. Lazarus, C. Velloso, P. Francis-West, G. M. Ellison-Hughes, S. D. R. Harridge

**Affiliations:** 1 Centre of Human and Aerospace Physiological Sciences, King’s College London, London, UK; 2 Stem Cell Institute, King’s College London, London, UK; 3 Department of Craniofacial development and stem cell biology, King’s College London, London, UK; 4 Cambridge Stem Cell Institute, University of Cambridge, Cambridge, UK

## Abstract

Wnt-β-catenin signalling is essential for skeletal muscle myogenesis during development, but its role in adult human skeletal muscle remains unknown. Here we have used human primary CD56^Pos^ satellite cell-derived myogenic progenitors obtained from healthy individuals to study the role of Wnt-β-catenin signalling in myogenic differentiation. We show that dephosphorylated β-catenin (active-β-catenin), the central effector of the canonical Wnt cascade, is strongly upregulated at the onset of differentiation and undergoes nuclear translocation as differentiation progresses. To establish the role of Wnt signalling in regulating the differentiation process we manipulated key nodes of this pathway through a series of β-catenin gain-of-function (GSK3 inhibition and β-catenin overexpression) or loss-of-function experiments (dominant negative TCF4). Our data showed that manipulation of these critical pathway components led to varying degrees of disruption to the normal differentiation phenotype indicating the importance of Wnt signalling in regulating this process. We reveal an independent necessity for active-β-catenin in the fusion and differentiation of human myogenic progenitors and that dominant negative inhibition of TCF4 prevents differentiation completely. Together these data add new mechanistic insights into both Wnt signalling and adult human myogenic progenitor differentiation.

## Introduction

Canonical Wnt signalling acting via the transcriptional co-activator β-catenin, is known to be critical for skeletal muscle myogenesis during embryonic development^1–3^. In the absence of Wnt ligands, a cytoplasmic ‘destruction complex’ maintains the cellular pool of β-catenin at a low level. A key action of this complex is to immobilise β-catenin providing glycogen synthase kinase 3β (GSK3β) the opportunity to phosphorylate it on key N-terminal serine and threonine residues, thereby marking β-catenin for degradation via the ubiquitin-proteasome pathway. Conversely, when Wnt ligands are present, this complex is inhibited and β-catenin remains in its active, unphosphorylated, form, which can translocate to the nucleus and regulate Wnt responsive genes via its partnership with TCF/LEF transcription factors^4^.

Little is known about the role of β-catenin in adult skeletal muscle, where it is assumed that many aspects of muscle development are recapitulated by the resident progenitor cell population, known as satellite cells, upon their activation in response to damage^5^. In resting muscle, undifferentiated satellite cells exist in a quiescent state beneath the basal lamina and directly adjacent to the terminally differentiated syncytial myofibres. In many species these dormant satellite cells are most commonly distinguished by their characteristic expression of the paired homeobox transcription factor 7 (Pax7); however, unlike in mice, human satellite cells can also be identified by their cell surface expression of CD56 (N-CAM)^6^. When muscle is damaged, satellite cells exit quiescence and sequentially express a well characterised cascade of myogenic regulatory factors, which in turn drive the expression of muscle specific genes. Two of these factors Myf5 and MyoD, are expressed immediately post-activation as the cells undergo proliferative expansion as myogenic progenitors, whilst myogenin and myogenic regulatory factor (MRF) govern the differentiation of these cells as they work to repair or replace damaged myofibres^5,7^.

The overwhelming majority of studies investigating the role of canonical Wnt-β-catenin signalling in skeletal muscle have been performed using cultured mouse cell lines. Manipulation of β-catenin levels in these lines has been reported to both inhibit^8–10^ and promote^10–15^ myogenic differentiation. Studies focusing on primary cells from adult mouse muscle and also mature mouse muscle *in vivo*, indicate roles for Wnt-β-catenin signalling in the regulation of both myotube^16^ and myofibre hypertrophy^17^. Although Wnt signalling is believed to be conserved between species, differences exist in the way satellite cells are identified in mice and humans and there are also emerging differences in the behaviour of isolated satellite cell-derived myogenic progenitors^18^. These differences mandate the need for species-specific studies to identify the role of Wnt-β-catenin signalling in human muscle adaptation and repair. This is particularly important as dysregulation of Wnt signalling has been implicated in sarcopenia, the age-regulated loss of skeletal muscle mass^19^.

We have investigated the role of canonical Wnt signalling in early passage adult human myogenic progenitors and, in particular, whether it acts as a facilitator or inhibitor of differentiation. Using an immunomagentic isolation procedure^6,20^ in combination with small molecule inhibitors and high-efficiency lentiviral expression constructs, we manipulated three key nodes in the Wnt pathway: GSK3, β-catenin, and T cell factor (TCF4/TCF7L2)^21^, in bulk populations of purified CD56^Pos^ myogenic progenitors obtained from numerous healthy individuals at early passage, therefore mitigating the potential impact of extended tissue culture artifacts^22^.

Manipulation of the core canonical Wnt components we selected disrupted the normal differentiation of human myogenic progenitors indicating the importance of this pathway in the regulation of these cells. We found that active GSK3 is required for the fusion and differentiation of human myogenic progenitors and that its inhibition represses the normal onset of myogenin expression. We show that while differentiation is not enhanced by overexpression of β-catenin, prevention of canonical Wnt signalling during differentiation via disruption of the nuclear β-catenin-TCF transcriptional complex completely abolishes differentiation. Together these data show that in adult human primary myogenic progenitor cells Wnt signalling is a central regulator of myogenesis and is essential for *in vitro* differentiation.

## Results

### β-catenin expression in adult human skeletal muscle tissue and primary myogenic progenitors

**Figure 1:**
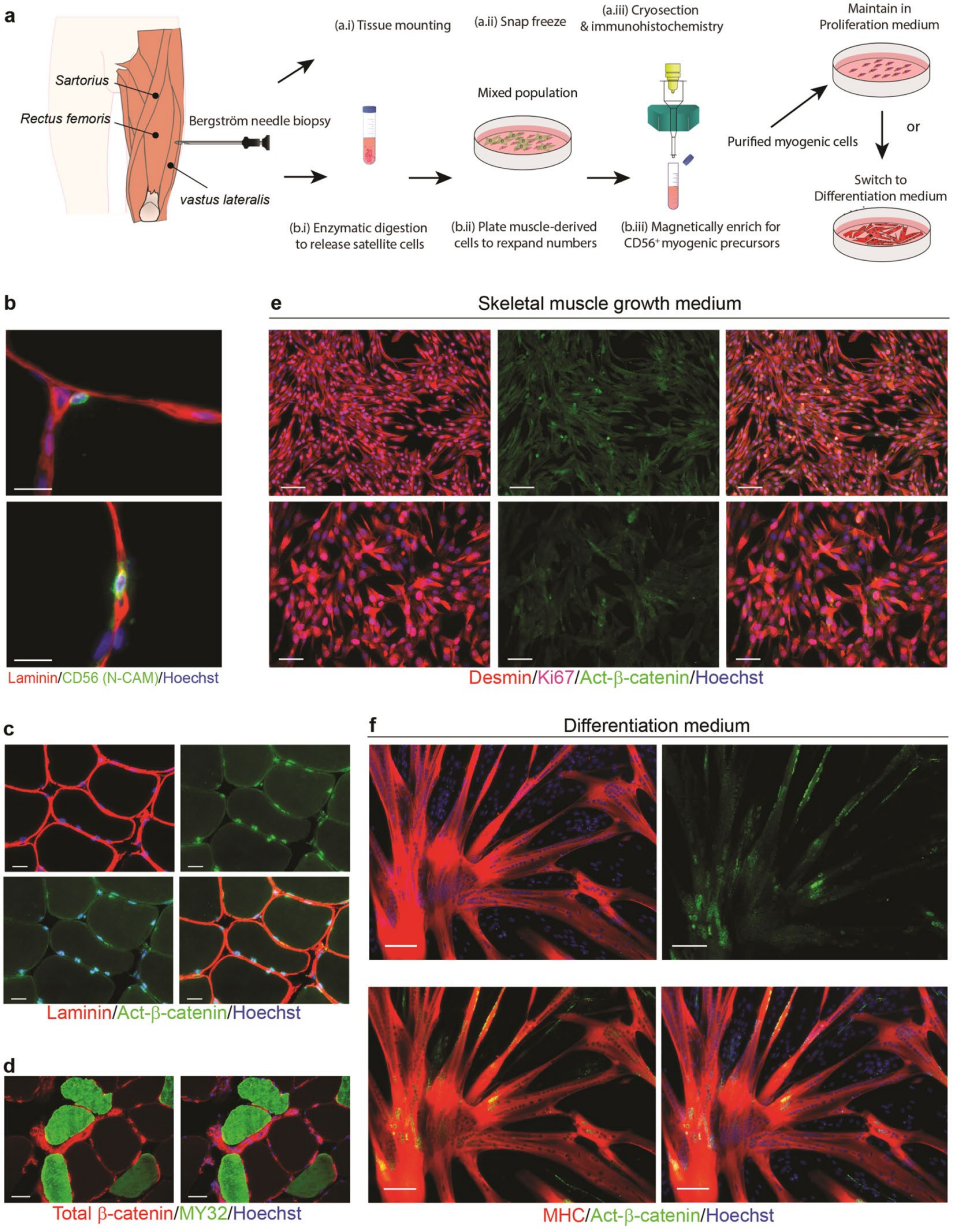
β-catenin expression in adult human skeletal muscle tissue and primary myogenic progenitors (**a**) Schematic of the experimental procedure whereby muscle tissue was obtained using the Bergström needle biopsy technique before being enzymatically digested to release myogenic cells. (**b**–**d**) Representative micrographs of healthy human muscle cryosections showed (**b**) CD56^Pos^ satellite cells, scale bar = 20 µm. (**c**) nuclear active-β-catenin expression in many satellite cells and (**d**) total-β-catenin expression localised at the periphery of many myofibers, scale bar = 20 µm. (**e**) Immunocytochemistry indicated an absence of nuclear active-β-catenin in proliferating desmin^Pos^/Ki67^Pos^ myogenic cells, scale bar = 100 µm (top), 50 µm (bottom) (**f**) Active-β-catenin was expressed in almost all nuclei of MHC^Pos^ multinucleated myotubes following 4 days of myogenic differentiation, scale bar = 100 µm. Assays for (**b**–**d**) performed on *n* = 3 biopsies; Staining for (**e**,**f**) was confirmed on *n* = 7 biopsies. All staining was performed with technical duplicates.

To assess the expression and localisation of β-catenin in adult human muscle, biopsy samples were taken from the vastus lateralis and either cryosectioned and analysed using immunohistochemistry, or enzymatically digested to allow immunomagnetic enrichment of CD56^Pos^, satellite cell-derived myogenic progenitor cells (Fig. 1a,b). Staining for active-β-catenin (non-phosphorylated)^23^ on cryosections of human muscle revealed distinct foci in many satellite cells, which can be distinguished based upon their location under the basal lamina (Fig. 1c). Staining for total-β-catenin was more readily detected in regions of cell-to-cell contact at the periphery of myofibres (Fig. 1d). To examine whether CD56^Pos^ satellite cell-derived human myogenic progenitor cells express active-β-catenin upon differentiation, immunomagentically purified primary cultures were established. We have previously shown that only cells within the CD56^Pos^ fraction of freshly isolated human muscle and not the CD56^Neg^ fraction have inherent myogenic capacity^6,20^. CD56^Pos^ cells are desmin^Pos^ and upon serum withdrawal readily form myotubes, which express myogenin﻿﻿﻿ and myosin heavy chain (﻿MHC)^6^. In proliferating CD56^Pos^/desmin^Pos^/Ki67^Pos^ myogenic progenitors, 8–9 days after isolation from human muscle at passage 1, β-catenin was detectable only at very low levels in the cytoplasm and there was no clear nuclear accumulation (Fig. 1e). Contrastingly, after four days of serum-free culture, which is a stimulus for myogenic differentiation^6^, these cells had terminally differentiated into large branching MHC^Pos^ multinucleated myotubes and the expression of cytoplasmic and nuclear active-β-catenin increased substantially (Fig. 1f). Together this indicates β-catenin is active in the quiescent progenitor cell population, downregulated in proliferating myoblasts and upregulated in differentiating myoblasts suggesting β-catenin signalling acts bi-phasically, either promoting or inhibiting myogenesis potentially depending on environmental signals and cell state.

### Receptor mediated canonical Wnt pathway activation does not affect myotube formation

**Figure 2:**
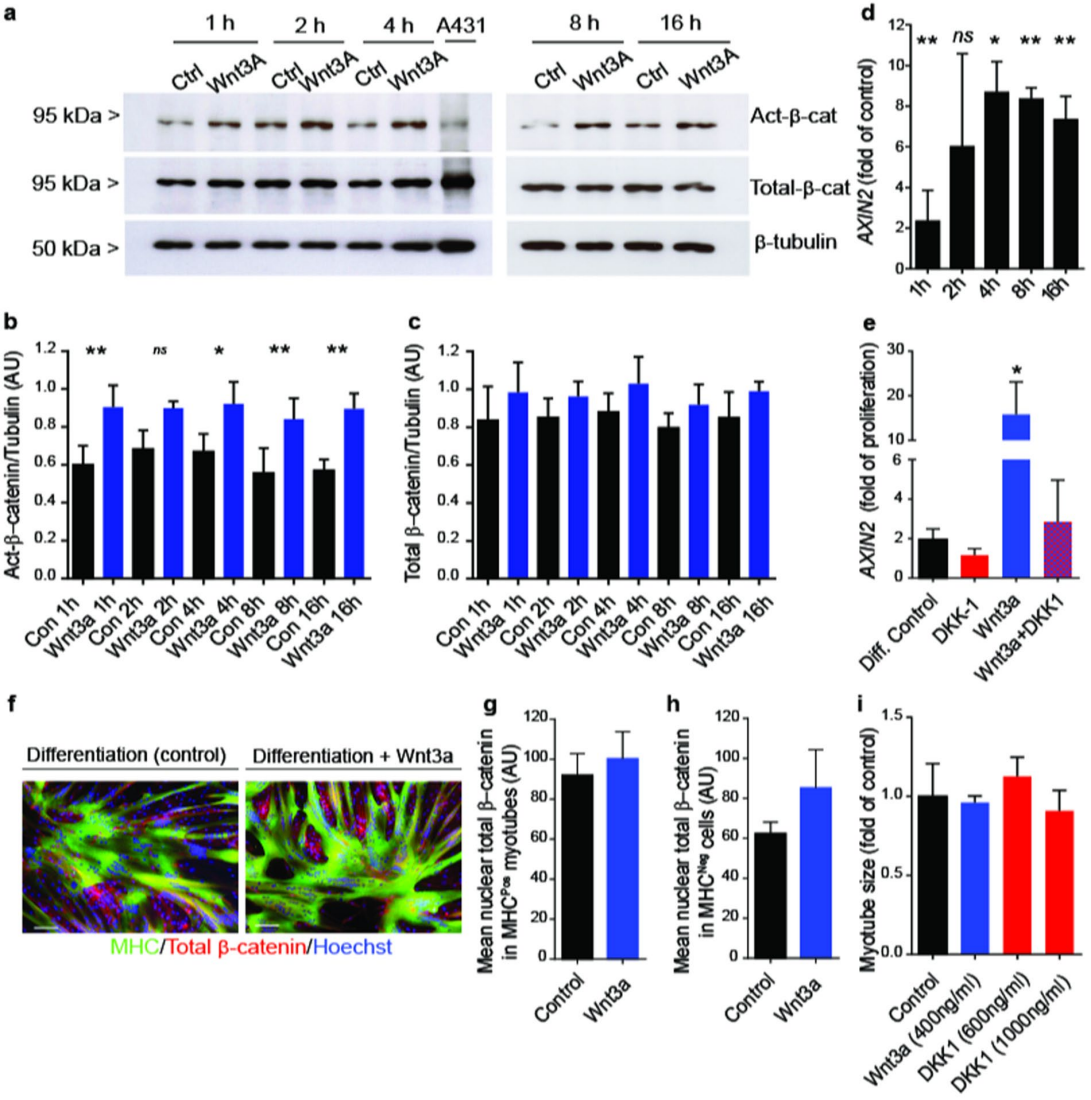
Receptor mediated canonical Wnt pathway activation does not affect myotube formation. (**a**) Western blot analysis of proliferating human myogenic cells treated with Wnt3a or control (0.3% BSA) for 1, 2, 4, 8 or 16 h. Equal protein loading was confirmed with β-tubulin immunoblotting and human epidermoid carcinoma cells (A431) were used as a positive control. (**b**) Immunoblot densitometry quantification of active-β-catenin/tubulin protein expression, *P < 0.05; **P < 0.01; ns: non-significant, (cells derived from *n* = 3 subjects). (**c**) Immunoblot densitometry quantification of total-β-catenin/tubulin protein expression, (cells derived from *n* = 3 subjects). (**d**) RT-PCR analysis of *AXIN2* expression following Wnt3a-treatment of human myogenic cultures, *P < 0.05; **P < 0.001; ns: non-significant (cells derived from *n* = 3 subjects). (**e**) qRT-PCR analysis of *AXIN2* expression in human myogenic progenitors cultured in differentiating conditions for 48 h  with Wnt3a, Dkk-1 or a combination of both relative to proliferating control, *P* < 0.05 (cells derived from *n* = 3 subjects). (**f**) Immunocytochemistry of total-β-catenin expression in control and Wnt3a-treated cultures (4 days). (**g**) Immunofluorescent quantification of nuclear total-β-catenin in MHC^Pos^ myotubes in control and Wnt3a-treated cultures (4 days), scale bars = 100 µm. (**h**) Immunofluorescent quantification of nuclear total-β-catenin in unfused MHC^Neg^ cells in control and Wnt3a-treated cultures (24 h). (**i**) Immunofluorescent quantification of mean myotube size in control Wnt3a or Dkk-1-treated myogenic cells (24 h). All bar charts are mean ± SD. For quantitation of immunofluorescence 6–10 independent fields of view were analysed per condition from *n* = 3 representative myogenic cell populations.

We sought to activate/inhibit Wnt signalling via the frizzled/LRP co-receptor complex to see if we could manipulate myotube formation. To achieve this, we supplemented the canonical Wnt ligand, Wnt3a, to the growth medium of human muscle-derived populations obtained from three separate individuals. As anticipated, western blotting showed that the addition of soluble Wnt3a induced a significant increase in active-β-catenin levels, while total-β-catenin was unchanged (Fig. 2a–c). Wnt stimulated primary cultures also demonstrated a strong and reproducible induction of the canonical Wnt target gene *AXIN2* within 4 hours of Wnt stimulation, which remained elevated for the remaining 16 hour time course (Fig. 2d). These data combined with the detection of other pathway components by RT-PCR demonstrate an active and responsive canonical pathway in human myogenic progenitors (Figure S1). In addition, when profiling differentiating human myogenic cells against a panel of known mammalian Wnt ligands, of the 10 canonical Wnts detected by qRT-PCR, Wnt3a was one of the most highly expressed (Fig. S1). To test that Wnt3a was activating β-catenin through its cell surface receptors we added Dkk-1, which is known to antagonise Wnt signalling by competing with the LRP receptor^24^. Addition of Dkk-1 was able to significantly reduce the Wnt3a-stimulated increase in *AXIN2* transcript expression, confirming the specificity of Wnt activation (Fig. 2e).

To assess whether receptor mediated stimulation of Wnt signalling would alter the differentiation of freshly isolated human myogenic progenitors, primary CD56^Pos^/desmin^Pos^ cultures were established and then switched to differentiation medium with the addition of Wnt3a at passage 2. After four days in serum-free conditions, both control and Wnt3a-treated cultures had differentiated into myotubes; furthermore the myotubes that developed in the presence of Wnt3a were visually indistinguishable from control myotubes (Fig. 2f). In both conditions myotubes strongly expressed MHC, a marker of terminal myogenic differentiation (Fig. 2f). Immunofluorescent quantification revealed that total-β-catenin expression within myotubes and also in undifferentiated mononuclear cells located outside myotubes remained comparable between control and Wnt3a cultures (Fig. 2g,h). Importantly, at the doses administered here, myotube area was unaffected by Wnt3a or Dkk-1, despite the ability of these factors to stimulate and inhibit Wnt signalling, respectively (Fig. 2i). Similarly, immunocytochemistry of Dkk-1 treated cultures confirmed comparable total-β-catenin and MHC expression (Fig. S1) despite a dose dependent reduction in active-β-catenin expression relative to control (Fig. S1). A TOP/FOP FLASH assay, which measures TCF transcriptional activity, was performed to ascertain the level of secreted canonical Wnt ligands in differentiating myogenic cell conditioned media collected between day 2–3 of differentiation. In this reporter assay, the TOP-FLASH plasmid luciferase is driven by functional TCF binding sites whereas the FOP-FLASH plasmid has mutated, non-functional TCF binding sites and serves as a negative control. The TCF transcriptional activity is shown as a ratio of TOP-FLASH to FOP-FLASH luciferase-mediated signals. This assay detected secreted canonical Wnts in all differentiating cultures obtained from different subjects, (n = 3) (Fig. S2).

These results suggest that while both Wnt3a and Dkk-1 modulate key components of this signalling pathway, neither factor alone is able to substantially effect myogenic differentiation. Whilst we were able to increase and decrease Wnt signalling via recombinant ligands acting at the level of cell surface receptors, it is wholly possible that the cells were able to constrain the strength of the Wnt signalling to within a homeostatic limit via the regulation of pathway components. Indeed, the increases in active-β-catenin protein when Wnt3a was added were modest and β-catenin remained readily detectable in the presence of Dkk-1.

### Active GSK3β is required for the fusion and differentiation of human CD56^Pos^ myogenic progenitor cells

In order to overcome potential autoregulation of the Wnt-β-catenin pathway we sought to experimentally increase cystolic β-catenin in adult human myogenic progenitors via small molecule inhibition of GSK3β, a core component of the canonical Wnt pathway and an essential regulator of β-catenin stability. We studied the effects of the GSK3α/β inhibitor, BIO^25^, on the progression of myogenic differentiation at 24 hours and 4 days following serum withdrawal corresponding to our definition of early and late stages of differentiation, respectively. These time points were selected as they allowed us to study both the initial stages of myogenic progenitor cell fusion and later their terminal differentiation into multinucleated myotubes.

**Figure 3:**
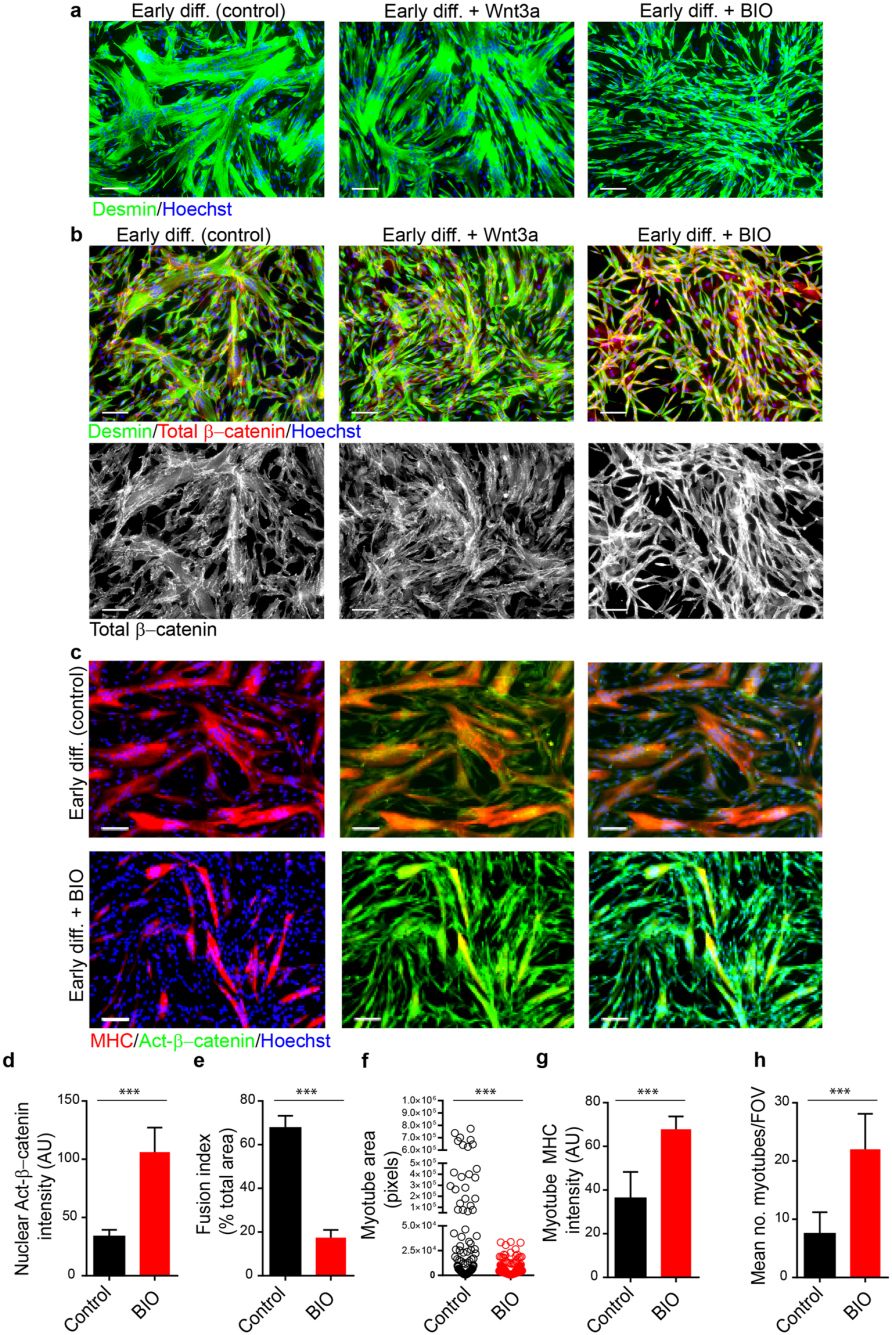
Active GSK3β is required for the fusion and differentiation of human CD56^Pos^ myogenic progenitor cells. (**a**) Representative micrographs of desmin^Pos^ human myogenic cells 24 h after induction of differentiation in control, Wnt3a and BIO-treated cultures. (**b**) Immunocytochemistry of total-β-catenin expression in myogenic cells exposed to control, Wnt3a and BIO-supplemented differentiation media. (**c**) Representative micrographs of active-β-catenin and MHC immunostaining of control and BIO-treated human myogenic cells (24 h). (**d**) Immunofluorescent quantification of nuclear active-β-catenin in control and BIO-treated cultures (24 h). (**e**) Immunofluorescent quantification of myotube fusion index in control and BIO-treated cultures (24 h). (**f**) Immunofluorescent quantification of mean myotube area in control and BIO-treated cultures (24 h). (**g**) Immunofluorescent quantification of myotube MHC fluorescent intensity in control and BIO-treated cultures (24 h). (**h**) Immunofluorescent quantification of the mean number of myotubes per field of view in control and BIO-treated cultures. All bar charts are means ± SD. For quantitation of immunofluorescence 6–10 independent fields of view were analysed per condition from *n* = 3 myogenic cell populations, ***P < 0.001 compared to control. All scale bars = 100 µm.

In stark contrast to both control and Wnt3a-treated cultures, which showed evidence of fusion and myotube formation at 24 hours under differentiating conditions, BIO-treated cultures showed very poor fusion with most cells remaining as mononucleated desmin^Pos^ progenitors (Fig. 3a). Although BIO-treated cultures showed some evidence of cell alignment (Fig. 3a), these cells exhibited a far less pronounced sequestration of total-β-catenin at the intracellular plasma membrane where β-catenin is known to play its crucial role in adherens junction formation^8^; instead cells cultured with BIO expressed total-β-catenin abundantly throughout the cytoplasm (Fig. 3b, Fig. S3).

In control cultures, after 24 hours in differentiation medium, nuclear accumulation of active-β-catenin could be observed within MHC^Pos^ cells (Fig. 3c, Fig. S4). However in BIO-treated cultures, myogenic fusion index was heavily depressed and quantification of nuclear fluorescence intensity revealed that these cells expressed more than twice the amount of nuclear active-β-catenin as measured in control conditions (Fig. 3c–e). Whilst myotube size was reduced in BIO-treated cultures (Fig. 3c and f; Fig. S4), the number of small MHC^high^ cells per field of view was significantly increased (Fig. 3﻿g﻿,h). Therefore taken together BIO-treated populations had increased expression of both total-and active-β-catenin, which led to an abundance of small MHC^High^ myotubes, which had failed to achieve their full potential for myogenic fusion suggesting a deregulation of the myogenic regulatory program upon GSK3β inhibition.

### GSK3β inhibition strongly inhibits the onset of myogenin expression under differentiating conditions

**Figure 4:**
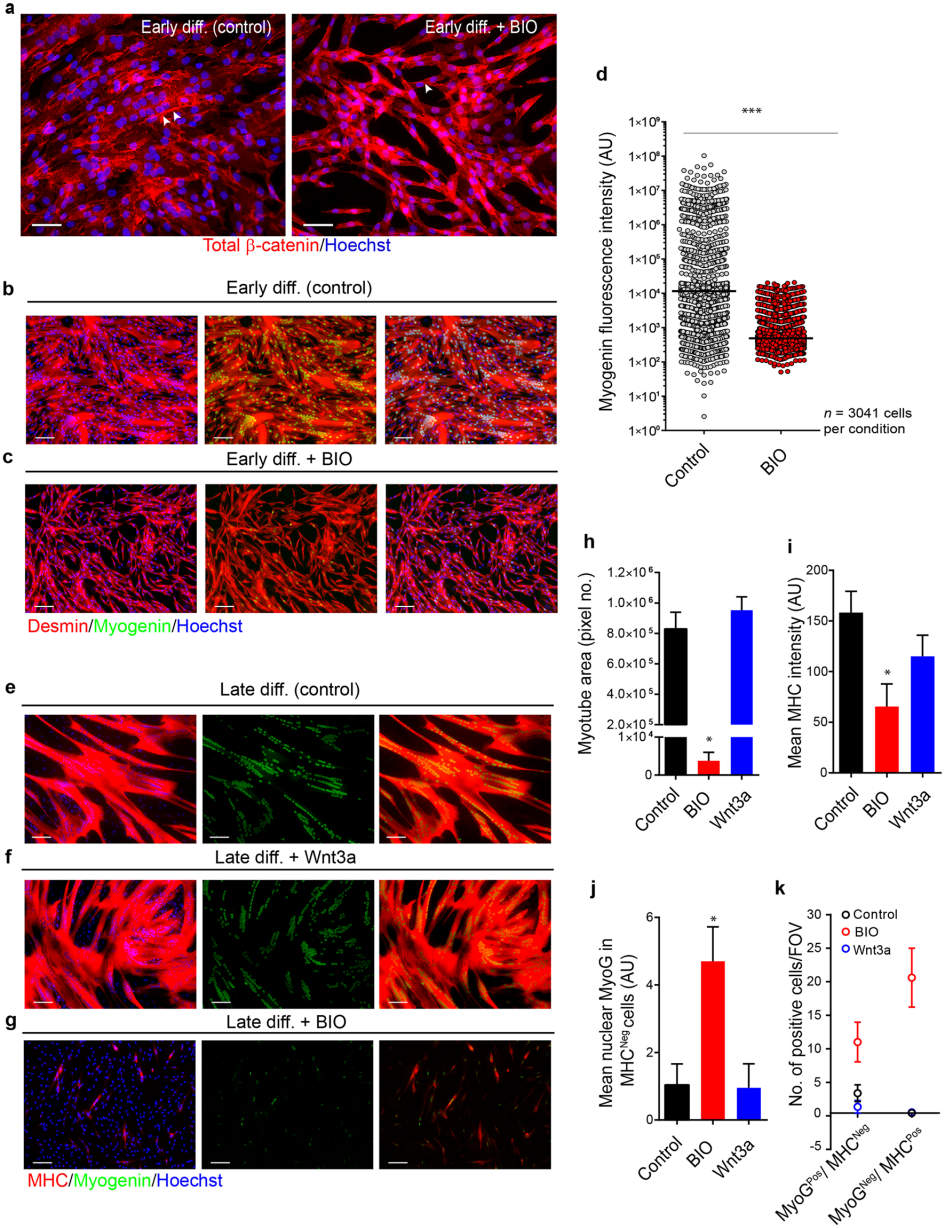
Myogenin expression is inhibited in human myogenic cells following GSKβ inhibition. (**a**) Representative micrographs of control myogenic cultures show clear accumulation of total-β-catenin (arrowheads) at the periphery of fusing myotubes while BIO-treated cultures display noticeably less junctional total-β-catenin staining (24 h). (**b**) Representative micrographs of m﻿yogenin expression in desmin^Pos^ human myogenic cultures in differentiation media (24 h). (**c**) Representative micrographs of ﻿myogenin expression in desmin^Pos^ human myogenic cultures after treatment with BIO for 24 h. (**d**) Quantification of nuclear myogenin fluorescent intensity in individual cells in control and BIO-treated myogenic cultures (24 h), ***P < 0.001; (**e**) Representative micrograph of myogenin expression in human myogenic cells differentiated for 4 days. (**f**) Representative micrograph of myogenin expression in myogenic cultures subjected to differentiation conditions in the presence of Wnt3a (4 days). (**g**) Representative micrograph of myogenin expression in myogenic cultures subjected to differentiation conditions in the presence of BIO (4 days). (**h**) Quantification of mean myotube area in control, BIO and Wnt3a-treated cultures. (**i**) Quantification of mean MHC fluorescent intensity in control, BIO and Wnt3a-treated cultures. (**j**) Quantification of nuclear myogenin in MHC^neg^ cells only in control, BIO and Wnt3a-treated cultures. (**k**) Quantification of myogenin^Pos^/MHC^Neg^ and myogenin^Neg^/MHC^Pos^ cells. All bar charts are means ± *SD*. In all cases *Indicates *P* < 0.05 compared to both control and Wnt3a-treated human myogenic cultures; analysis was performed on *n* = 4 independent myogenic cell populations. Scale bars = 100 µm.

In control human myogenic populations cultured in differentiating conditions for 24 hours, sites of cell-to-cell contact showed a high level of total-β-catenin expression (Fig. 4a). In contrast, BIO-treated cells showed a far less robust accumulation of total-β-catenin at cell-cell contact sites, but cytoplasmic and nuclear levels were far higher than that observed in control conditions (Fig. 4a). To check whether this mislocalisation of total-β-catenin might impact upon the core transcription factor network governing myogenesis, we assessed the localisation and expression of myogenin, a key transcriptional regulator of myogenic differentiation^26^. This transcription factor could be clearly identified in the nuclei of differentiating human myogenic progenitors after 24 hours of serum withdrawal (Fig. 4b). However, consistent with the stunted myotube differentiation in BIO-treated cultures, myogenin expression was heavily suppressed in these cultures when compared to untreated control populations (Fig. 4c). Cell-by-cell analysis of myogenin immunofluorescent intensity further confirmed a large and highly significant reduction in nuclear myogenin levels under conditions of GSK inhibition (Fig. 4d). In contrast, early differentiation in the presence of Wnt3a did not significantly prevent the upregulation of myogenin and myotube formation was highly comparable to control populations (Fig. S2). Thus at this early time point of differentiation, these data suggest that additional stimulation of receptor mediated Wnt signalling does not abrogate myogenic differentiation, whereas small molecule disruption of GSK3β activity has a profoundly inhibitory impact on myogenin expression under differentiating conditions (Fig. 4b–d).

To see whether myogenic differentiation is simply delayed when GSK3β is constitutively inhibited, we maintained human myogenic cells until day four (late differentiation) with or without Wnt3a or BIO supplementation. By late differentiation, control populations differentiated into large, multinucleated myotubes and the nuclei within these MHC^Pos^ structures stained strongly for active-β-catenin (Fig. S3). Quantification of fusion index showed that while >70% of cells in both control and Wnt3a-treated cultures were incorporated into myotubes after four days <10% of myogenic cells had fused in BIO supplemented cultures (Fig. S3). This lack of fusion cannot be attributed to a loss of myogenic fate amongst CD56^Pos^ derived progenitors as the unfused MHC^Neg^ cells remained as desmin^Pos^ mononuclear cells (Fig. S3); nor can it be attributed to differences in cell density as the total nuclear area per field of view between control and BIO cultures was not significantly different between the three culture conditions (data not shown). In line with the successful terminal myogenic differentiation in control and Wnt3a-supplemented cultures, all nuclei within myotubes ubiquitously expressed myogenin at day 4 of differentiation (Fig. 4e,f). Contrastingly, there was a large reduction in myogenin expression in BIO-treated populations (Fig. 4g; Fig. S5) along with a significant reduction in myotube area (Fig. 4h) and MHC immunofluorescent intensity (Fig. 4i).

Notably, under control differentiation conditions there was almost no expression of myogenin in cells located outside myotubes (MHC^Neg^), which are comprised of myogenic cells that have failed to differentiate (including reserve cells) (Fig. 4j,k; Fig. S5). However, BIO supplemented cultures showed evidence of atypical myogenin and MHC expression (Fig. 4j,k; Fig. S5) as evidenced by a significant increase in myogenin^Pos^/MHC^Neg^ cells and to a greater extent MHC^Pos^/myogenin^Neg^ cells in BIO cultures (Fig. 4j,k; Fig. S5). In order to confirm that ﻿myogenin is directly modulated by BIO we allowed myogenic cultures to differentiate for 2 days so that they started to fuse, forming myogenin^Pos^ myotubes before applying BIO for a further 24 h. Only in BIO-treated cultures did we observe a significant reduction in myogenin expression, indicating a direct modulation of myogenin by BIO, independent of fusion (Fig.S5).

In order to assess whether BIO-mediated disruption of myogenin expression was due to myogenic cells being stalled in a proliferative state, we assayed the percentage of Ki67^Pos^ cells in proliferation media (24 h), differentiation media and differentiation media supplemented with BIO (72 h). Immunostaining revealed that as expected myogenic cells maintained in proliferation media were almost exclusively Ki67^Pos^/myogenin^Neg^ while cells maintained in differentiation media contained a high percentage of myogenin^Pos^ cells and lacked discernible expression of Ki67 (Fig. S6). Interestingly, cultures treated with BIO had significantly more Ki67^Pos^ cells and lacked notable myogenin expression. Therefore, BIO-mediated disruption of myogenin is accompanied by an increase in myogenic cells stalled in a proliferative state.

To test that the differentiation defects in BIO cultures were not a result of non-specific effects of the BIO compound itself, we performed the same experiments using meBIO, its kinase inactive form (Fig. S7). Myogenic cells cultured with meBIO differentiated to the same extent as control populations and expressed an identical level and localisation of active-β-catenin and myogenin (Fig. S7). To explore the specificity of the role of GSK3β in the disrupted myogenic differentiation we repeated the differentiation process with two further inhibitors of GSK3β, which are structurally diverse, CHIR and LiCl (Fig. S7 and S8). Treatment with both CHIR (Fig. S7) and LiCl (Fig. S8) stabilised β-catenin while concomitantly suppressing myogenin expression and stunting myotube formation.

Taken together these series of experiments demonstrate that the pharmacological inhibition of GSK3β markedly impairs the fusion and differentiation of primary adult human muscle progenitor cells through the deregulation of myogenin expression.

### The β-catenin-TCF transcriptional partnership is essential for the fusion and differentiation CD56^Pos^ human myogenic progenitors

**Figure 5:**
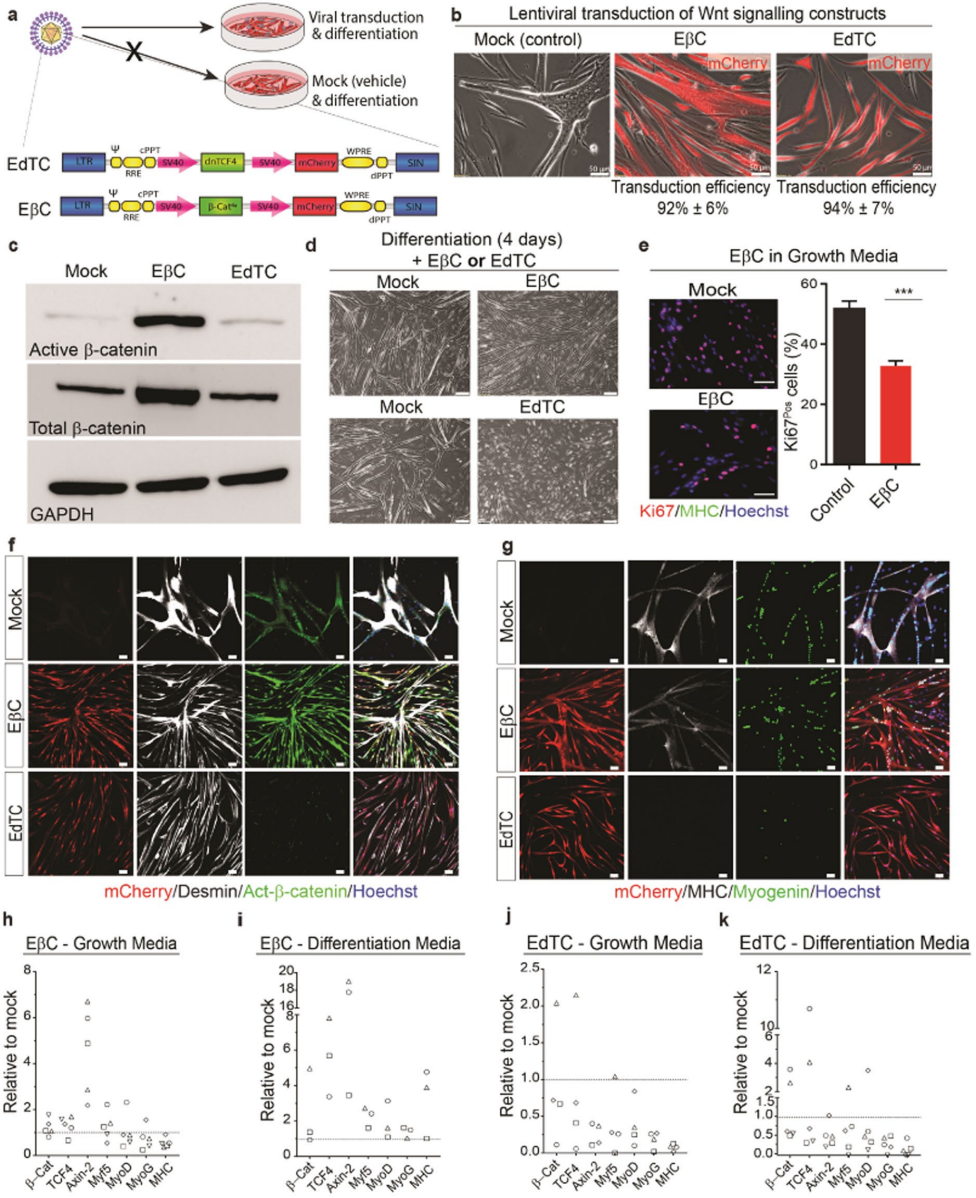
Lentiviral manipulation of Wnt-β-catenin signaling. (**a**) Schematic of the lentiviral constructs. (**b**) Representative micrographs of mock, EβC and EdTC-transduced cells, scale bar = 50 µm. (**c**) Western immunoblotting for active-β-catenin and total-β-catenin in mock, EβC and EdTC-transduced cells. (**d**) Representative micrographs of mock, EβC and EdTC-transduced human myogenic cultures after 4 days in differentiation media, scale bar = 200 µm. (**e**) Ki67/MHC immunostaining of mock and EβC human myogenic cells in proliferative conditions (24 h), scale bar = 100 µm. (**f**) Desmin/active-β-catenin immunostaining of mock, EβC and EdTC human myogenic cultures. (**g**) MHC/myogenin immunostaining of mock, EβC and EdTC human myogenic cultures (4 days), scale bar = 50 µm. (**h–k**) qRT-PCR analysis following of EβC/EdTC myogenic cells cultured in either growth or differentiation media for 48 hours, each symbol represents individual cultures derived from different biopsies and shows gene expression relative to mock control from the same individual, (*n* = 3–5).

To test whether the greatly increased levels of β-catenin in GSK3β inhibited cultures were directly responsible for the suppression of myogenin and lack of fusion, we transduced purified CD56^Pos^ human myogenic progenitors with a lentiviral expression construct to induce the overexpression of active-﻿β-catenin (EβC) which cannot be targeted for degradation by GSK3-mediated phosphorylation. In addition, to examine the necessity of the transactional co-activator role of β-catenin in human myogenic cell differentiation we also transduced purified primary cell lines with a construct overexpressing a dominant negative form of TCF4 (EdTC). This dominant negative mutant form of TCF4 harbours an N-terminal deletion, such that it binds Wnt target genes, but cannot transactivate them due to its inability to bind β-catenin^21,27^. In both cases successful integration was identified by mCherry expression (Fig. 5a,b). Fluorescent microscopy was used to confirm a reproducible rate of transduction, which in all cases exceeded 92% of the population (Fig. 5b). Overexpression of active-β-catenin in EβC transduced cells was confirmed by both western blot and immunofluorescence analysis (Fig. 5c). EdTC overexpression on the other hand, had little impact on β-catenin expression, which is consistent with its mechanism of action (Fig. 5c). Observation of EβC-treated cultures revealed a reduction in myogenic fusion, while EdTC cultures showed a much more severe failure of differentiation (Fig. 5d).

To assess whether β-catenin might exert a pro-differentiation effect, as has been suggested for mouse myogenic cells^12^, we cultured EβC-transduced cells in growth medium conditions. Interestingly, EβC significantly decreased the percentage of Ki67^Pos^ actively proliferating cells, but in the absence of differentiating conditions did not drive myogenic differentiation confirmed by a lack of MHC expression in both EβC and control myogenic cells when cultured in growth media (Fig. 5e).

EβC overexpression in differentiating conditions resulted in easily identifiable increases in nuclear active-β-catenin and MHC^Pos^ myotubes were evidently thinner than their autologous control counterparts. Nuclear myogenin was also found to be reduced in EβC cultures (62% ± 8%) compared to control cultures (74% ± 4%) (Fig. 5f,g). In aggregate, this suggests that β-catenin alone is not sufficient to drive differentiation while increases above a pre-defined range can negatively affect differentiation. Interestingly EdTC-transduced cells had drastically reduced expression of active-β-catenin, were extremely thin, did not undergo fusion or differentiation and did not express any detectable levels of myogenin or MHC protein (Fig. 5d and f,g), suggesting an active-β-catenin-TCF complex in human myogenic differentiation is essential.

**Figure 6:**
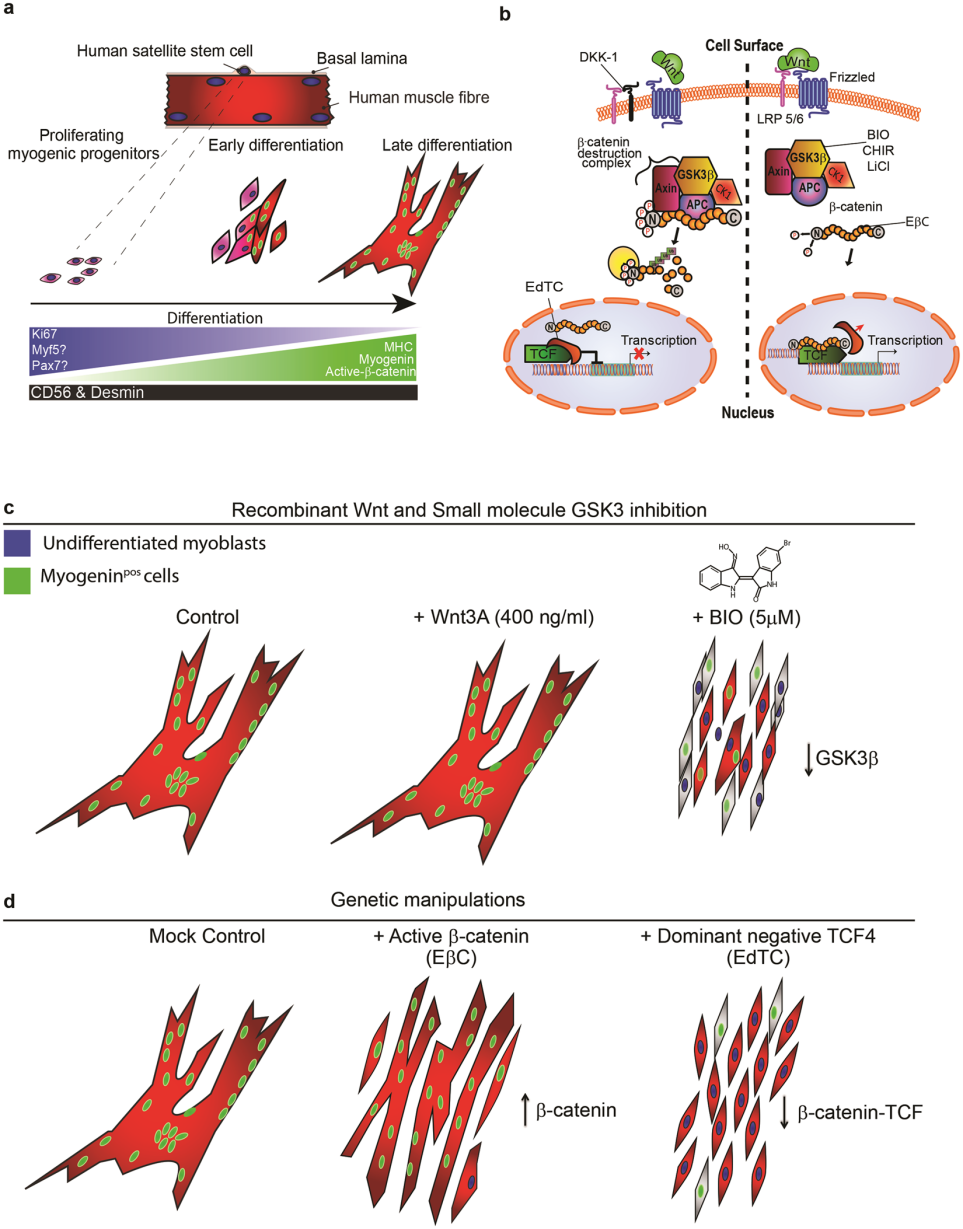
Active GSK3β is an absolute requirement for the terminal differentiation of human myogenic progenitor cells. (**a**) Serum withdrawal stimulates myogenic cells to align, fuse and differentiate to form multinucleated myotubes. After 24 h active-β-catenin expression had become localised to the nucleus of myogenic cells, which had begun to align/fuse. Continued mitogen withdrawal for 4 days resulted in the formation of terminally differentiated myotubes, which were multinucleated and expressed the terminal differentiation markers myogenin and MHC. (**b**) Graphical illustration shows the key nodes of the Wnt pathway that were perturbed. (**c**) Treatment of myogenic populations with GSK3β inhibitors markedly reduced myogenic cell fusion, myotube formation and myogenin expression compared to control and Wnt3a supplemented cultures. (**d**) Genetic manipulation with lentiviral vectors to overexpress active-β-catenin (EβC) or dominant negative TCF4 (EdTC) similarly reduced the capacity of human myogenic cells to undergo myogenic differentiation. EβC had marked effects on the completion of myogenic differentiation while EdTC lead to a robust inhibition of myogenic differentiation thus uncovering a crucial role for this core canonical Wnt signalling transcription factor in the regulation of human myogenic differentiation.

To uncover the molecular mechanisms underpinning the phenotypes observed, we performed qRT-PCR analysis on transduced cells maintained in either proliferating or differentiating conditions. In Fig. 5h–k, each symbol represents a different individual and their subsequent readout for the corresponding gene. This analysis revealed that overexpression of β-catenin (EβC) reproducibly led to increased *AXIN-2* expression relative to mock control, however, a variable response was noted depending on the individual, ranging from a moderate, 2–7 fold, increase to a very high, 17–19 fold, increase. EβC-transduced cells cultured in growth medium underwent active suppression of myogenic regulatory genes, with the exception of Myf5 (Fig. 5h). This supports the contention that canonical Wnt signalling alone does not induce differentiation in human myogenic cells in growth conditions (Fig. 5h). Analysis of EβC cultures in differentiation medium confirmed a moderate but variable upregulation of *β-CATENIN* and *TCF* (endogenous form), *MYF5*, *MYOD*, and *MHC* transcripts when compared to mock transduced controls (Fig. 5i). Quantitative RT-PCR analysis of TCF4 dominant negative (EdTC) cultures after 48 hours in either proliferating or differentiating conditions all showed decreased expression of *AXIN-2* along with significantly reduced levels of *MYOGENIN* and *MHC* transcripts relative to mock control (Fig. 5j,k). Taken together this shows that an optimal level of β-catenin is required for efficient human myogenic cell fusion and differentiation while an intact β-catenin-TCF complex is essential (Fig. 6).

## Discussion

Wnt proteins and their signalling pathways have been the subject of intense investigation in recent years owing to their important roles in a vast array of developmental, homeostatic and disease processes^28–32^. Of the three main branches of Wnt signalling, the canonical pathway, which is dependent on β-catenin, is essential for the formation of skeletal muscle during embryogenesis^4^. Despite the necessity of this pathway for developmental myogenesis, there has been considerable controversy surrounding the actions of β-catenin in adult mouse muscle^17,33–35^, while its role in human muscle remains completely uncharacterised.

In the present work we manipulated the activity and/or level of three key nodes in the Wnt signalling pathway, GSK3β, β-catenin and TCF4/*TCF7L2* in primary human myogenic progenitor cells^6^, in order to identify possible roles for this pathway in the regulation of myogenic differentiation. Our data revealed that manipulation of Wnt signalling outside of pre-defined limits were detrimental to human *in vitro* myogenesis. Throughout this process we revealed a previously unidentified necessity for active GSK3β in the fusion and efficient terminal differentiation of human myoblasts via its regulation of myogenin.

The demonstration of abundant protein expression of both nuclear active and total forms of β-catenin in resting adult human skeletal muscle *in situ* indicates that the Wnt-β-catenin pathway is operational *in vivo* (Fig. 1). We next sought to examine a possible role for Wnt signalling in an *in vitro* model of regenerative myogenesis^6,20^. In purified populations of CD56^Pos^ myogenic progenitor cells we demonstrated that the nuclear levels of active-β-catenin in proliferating progenitors were low. We define high nuclear accumulation as the point at which the nucleus of cells could be demarcated by positive beta-catenin staining alone, without the need to refer to Hoechst. However, after 24 hours of serum withdrawal, a trigger for myogenic differentiation, nuclear accumulation of active-β-catenin became readily upregulated (Fig. 1). By four days of serum-free culture almost every nuclei within terminally differentiated MHC^Pos^ myotubes expressed clear nuclear staining of active-β-catenin (Fig. 1). This is contrary to observations made by Otto *et al*. who identified the regulation of proliferation as the primary role of nuclear active-β-catenin in mouse satellite cells^30^. Our data is, however, consistent with the work of Brack *et al*. who also showed a marked increase in canonical Wnt signalling at the onset of differentiation^12^.

Given the proposed roles of Wnt-β-catenin signalling in regulating myogenic differentiation we sought to activate the canonical Wnt pathway by treating purified populations of myogenic progenitors with soluble recombinant Wnt3a (Fig. 2). Similar to previous studies we utilised both the stabilisation of β-catenin protein and *AXIN2* mRNA as readouts of active canonical Wnt signalling^12,36^ and confirmed a significant increase in active signalling following Wnt3a stimulation (Fig. 2a–e). We also showed that canonical pathway activation could be competitively inhibited using the Wnt signalling antagonist, Dkk-1^37^ (Fig. 2e). However, to our surprise, despite clear modulation of the pathway, neither Wnt3a nor Dkk-1 conferred any substantial effect on the successful completion of myogenic differentiation (Fig. 2f–i; Supplementary Figures S2 and S3). We postulated that the addition of Wnt3a may potentially have been unable to activate the canonical pathway in a sustained manner, owing to the reported presence of negative feedback loops that serve to regulate the intensity and duration of the Wnt signal^36,38^. *AXIN2*, a direct Wnt target and negative regulator, is known to augment the GSK3β mediated phosphorylation of β-catenin by several thousand fold, greatly increasing β-catenin degradation^39^. Our data show strong induction of *AXIN2* within 2 hours of Wnt3a administration supporting the activation of this negative feedback loop. In order to circumvent this issue we decided to stabilise β-catenin via pharmacological inhibition of GSK3β (GSKi) using three structurally diverse molecules (BIO, LiCL and CHIR^40^), all of which have been shown to activate canonical Wnt signalling in a variety of different systems. We confirmed the ability of these compounds to bypass AXIN-mediated negative feedback to stabilise the active form of β-catenin in our human myogenic progenitors (Fig. 3, 4,  S7 and S8).

In contrast to the lack of phenotype observed with the addition of soluble Wnt3a, GSKi via either BIO, CHIR, or LiCL led to a severe blunting of the normal differentiation response with significant reductions in fusion index, individual myotube size and overall MHC expression in the treated cultures. The pronounced loss of differentiation potential in GSKi cultures suggested a role for GSK3β activity in regulating MRFs, which are essential for skeletal myogenesis during embryonic development and for post-natal regenerative myogenesis^41^. Of the MRFs, myogenin is known to be critical for terminal differentiation^26,42^. Irrespective of the inhibitor used, we were able to show that GSKi was associated with a significant repression of myogenin (Fig. 4 and Fig. S7–S8). GSKi cultures also had an increased number of cells with mutually exclusive expression of myogenin and MHC, which were only rarely observed in control populations (Fig. 4 and S5). BIO was also shown to modulate expression of myogenin independent of fusion (Fig. S5). Interestingly, cultures treated with BIO for 72 hours in serum-free differentiation also had significantly more Ki67^Pos^ cells indicating that the cells were either continuing to proliferate or were stalled as progenitors (Fig. S6). However, as the number of cells/field of view did not increase or decrease over the culture period when compared to control populations, and there was no indication of increased cell death (Fig. S6), it appears that these cells were unable to progress beyond the progenitor phase.

This suggests that GSKi leads to a dysregulation in the typically co-ordinated sequence of MRF expression leading to failures in fusion and differentiation. Use of three structurally diverse inhibitors of GSK3β suggested that the phenotypes observed were based on loss of GSK3β activity as opposed to off target effects^43^. Furthermore, the observation of normal differentiation using meBIO, an almost structurally identical molecule to BIO, but lacking its ability to inhibit GSK3β^44,45^, ruled out the possibility of non-specific toxicity.

That GSKi and Wnt3a did not produce the same outcome for myogenic differentiation is perhaps surprising given that GSK3β inhibitors are often used as a proxy for canonical Wnts. At least some of this discrepancy may be attributable to the different mechanisms of action of the GSK3β inhibitors and Wnt proteins^46^. Inhibitors of GSK3β reduce the catalytic activity of GSK3β, which allows β-catenin to escape degradation. In comparison, the prevailing view is that binding of Wnts to their cell surface receptors does not affect the kinase activity of GSK3β, but rather blocks the destruction complex by sequestering it at the cytoplasmic tail of LRP at the cell membrane^46^. In comparison the reduced accumulation of β-catenin observed at cell-cell contact sites in GSKi conditions may have contributed to lower fusion^47^. Disruption of cell-cell cadherin ligation is known to abrogate myogenic cell fusion^48^ and previous authors have suggested a mechanism by which reductions in GSK-mediated cadherin phosphorylation can reduce its affinity for β-catenin^49,50^.

To ascertain whether the differentiation phenotype we observed was due solely to increased β-catenin expression and to overcome any concerns over the specificity of inhibitors of GSK3β, given the role of this kinase beyond canonical Wnt signalling, we used a highly efficient lentiviral-overexpression constructs containing an active form of β-catenin (EβC). Overexpression of active-β-catenin produced a phenotype of reduced fusion and myotube size, which was similar but less severe than that observed with GSKi (Fig. 5). This was likely due to maintained expression of myogenin in the case of EβC (Fig. 5), but not in GSKi conditions (Fig. 4). In turn this suggests that active GSK3β is required for the induction of myogenin independently of β-catenin.

Given that β-catenin is highly upregulated during normal myogenic differentiation we sought to determine whether β-catenin overexpression in proliferation conditions may stimulate myogenic differentiation. Rather this led to a moderate downregulation in MRFs (Fig. 5h) and did not stimulate myotube formation (Fig. 5e). In support of our data, Goichberg *et al*., also observed a reduction in myogenin following β-catenin overexpression from a retroviral vector in growth medium^8^. We did however uncover a reduction in the proportion of proliferating progenitors with EβC overexpression (Fig. 5e). Conversely, when conditions were permissive for differentiation, β-catenin overexpression led to moderate increases in expression of MRFs and MHC in two of the three individuals tested. Nonetheless, the myotubes that formed in EβC cultures were noticeably thinner and more numerous per field of view than control myotubes. Thus, in primary human myogenic cells it appears that β-catenin alone is not sufficient to drive differentiation in non-permissive conditions and that it likely interacts with other factors, which are dependent on the cellular context.

To examine the co-activator role of β-catenin in myogenic differentiation we overexpressed a dominant-negative mutant form of TCF4 (EdTC). EdTC is able to bind DNA at Wnt target genes, but cannot transactivate them due to its inability to bind β-catenin^51^. Schuijers *et al*.^27^ demonstrated that overexpression of dnTCF4 was sufficient to abolish almost all β-catenin/DNA interaction, elegantly confirming that canonical Wnt signalling is exclusively transduced via TCF transcription factors^27^. In our hands knockdown of TCF-driven transactivation severely disrupted differentiation (Fig. 5). In EdTC cultures, levels of all myogenic regulatory factors were significantly downregulated in both proliferation and differentiation conditions (Fig. 5). Furthermore, myogenin and MHC were greatly diminished at the protein level (Fig. 5g). Thus, we show here that β-catenin-TCF interactions are indispensable for human myogenic differentiation. As the same phenotype was observed across multiple separate transductions of primary cells from different human participants this strongly suggests that the findings were not attributable to disruptive positional effects of the construct integration. In line with our work, previous studies in mice have suggested that TCF4 might directly regulate MHC expression by direct interaction with the promoter/enhancer region of MHC genes^52–55^.

Recent work in mice whereby either the Wnt agonist BIO was administered or β-catenin was overexpressed specifically in satellite cells following muscle injury, demonstrate a striking phenotype of vastly protracted regeneration and the appearance of many small fibres^56^. This was also seen *in vivo* with β-catenin overexpression in mouse muscle^34^. Another study, which knocked out APC in mouse satellite cells leading to unrestrained β-catenin activation produced similar findings^57^. Recently, Rudolf *et al*. showed that loss of beta-catenin lead to protracted and incomplete regeneration but that over-expression of increased active beta-catenin resulted in precocious differentiation in mouse myogenic cells. These authors argued that both increased or loss of Wnt-β-catenin signalling leads to impaired *in vivo* muscle regeneration with decreases in satellite cell self-renewal, decreased fibre size and excess collagen deposition^10^. Collectively, these studies and ours suggest that the manipulation of β-catenin above or dramatically below physiological levels is broadly disruptive to myogenesis. In contrast, Murphy *et al*. utilised a Pax7-Cre driver and did not observe any phenotype from loss of β-catenin suggesting that it is dispensable for regenerative myogenesis in the adult^34^. However, because Cre-based recombination is imperfect, not all satellite cells will have deleted the gene entirely. In addition satellite cells are abundant in muscle with estimates of 5–7 per fibre; once activated they are highly proliferative and only a few cells are needed to regenerate and repopulate a large area of muscle^58^. If a knockout and wild type cell were able to fuse, β-catenin from the wild type cell could be shared rendering the knockout obsolete. Thus a very small number of ‘escaper cells’ with functional β-catenin could potentially compensate. As the level and localisation of β-catenin in this study was not quantified over time, the extent of β-catenin expression following the experimental manipulations remains unclear^34^.

We have demonstrated that canonical Wnt signalling is present in adult human skeletal muscle *in situ* and active in CD56^Pos^ myogenic progenitors *in vitro*. The present study provides evidence that β-catenin is dynamically regulated during the different phases of myogenesis increasing as differentiation progresses. Manipulation of key Wnt signalling components during differentiation has unique outcomes. GSKi leads to inhibition of myogenesis whilst Wnt proteins, which activate the signalling pathway (Fig. 2), have little effect; these data bring into question the use of GSKi as a proxy for Wnt signalling. Despite its onset in our *in vitro* differentiation paradigm, further increases in the amount of active-β-catenin in growth conditions does not promote differentiation, but rather inhibits it. This is evidenced by a trend towards lower expression of genes associated with myogenic differentiation and no increase in MHC or myogenin as detected by immunostaining (Fig. 5). On the other hand, during differentiation, myogenic genes tended to increase with β-catenin overexpression but this did not translate into better myotube formation; in fact, myotubes were very clearly thinner with an increased background of unfused cells (Fig. 5). Thus, our work demonstrates that β-catenin can exert opposing effects on myogenesis depending on whether cells are exposed to conditions, which favour proliferation or differentiation. In the knowledge that differentiation fails with the addition of dominant negative TCF it appears that, for optimal myogenic differentiation, a specific level of Wnt signalling is critical. An appreciation of how core components in the canonical Wnt pathway influences the progression of adult myogenesis may assist us in experimentally manipulating this pathway for therapeutic gain in conditions where muscle regeneration is compromised.

## Methods

### Cell isolation and culture

All studies were performed with UK National Health Service Ethics Committee approval and in accordance with the Human Tissue Act and Declaration of Helsinki. Using the Bergström needle biopsy technique^59^ with additional suction, muscle samples were obtained from the vastus lateralis of healthy young adult male volunteers (age 25.6 ± 3.5 years, height 179.0 ± 5.3 cm, weight 70.3 ± 8.6 kg) who gave written informed consent to participate in this study (*n* = 22). Muscle-derived cells were isolated, cultured for 5–7 days to expand numbers and immunomagnetically purified using anti-CD56 microbeads as described previously^6,20^. In all studies reported here primary human muscle-derived cells at passage 1 or 2 were used and at least 2–3 replicate wells were plated and analysed per condition.

After CD56^Pos^ purification, cells were plated on collagen-coated (0.5 mg/ml calf skin collagen, Sigma, USA) borosilicate glass coverslips (Ø 13 mm; no. 1 thickness, VWR, UK) in 24 well plates (ThermoScientific, Germany). For western blotting and qRT-PCR experiments, cells were plated at 40,000 cells/35 mm dish. For proliferation cells were plated at 10,000 cells/well in 24 well plates and for differentiation cells were plated at 25–50,000 cells/well. For all studies investigating differentiation the cell monolayer was rinsed three times with PBS prior to the switch from proliferation to differentiation medium to remove all traces of serum containing medium^60^. Full details of media conditions as described previously^6^.

### Snap freezing of human skeletal muscle

To characterize the expression of CD56 and β-catenin *in situ*, human skeletal muscle biopsies obtained from healthy individuals were snap frozen in dry ice-cooled isopentane and cryosectioned at 10 μm thickness (*n* = 2). Tissue sections were stained as detailed below.

### Pharmacological manipulation of Wnt-β-catenin signalling

6-bromoindirubin-3′-oxime (BIO) or its kinase inactive form 6-Bromo-1-methylindirubin-3′-oxime (meBIO) (Merck Chemicals, UK) were diluted to a final working concentration of 5 μM^46,61^, lithium chloride (LiCl) (Sigma, UK) was diluted to a final working concentration of 20 mM/L, CHIR99021(CHIR) (Stemgent, USA) was diluted to a final working concentration of 5 μM. Each was administered to cells at their working concentrations in an appropriate volume of serum-free differentiation medium. In all cases the final concentration of DMSO was <0.2% v/v. Recombinant proteins Wnt3a and Dkk-1 (R&D Systems Europe Ltd., UK) were reconstituted in sterile PBS containing 0.2% BSA. Proteins were administered at 200–400 mg/ml (Wnt3a) and 600–1000ng/ml (Dkk-1) in serum-free skeletal muscle differentiation medium containing 0.3% (w/v) BSA as a carrier protein.

### TOP/FOPflash Assay

Dual Luciferase assay kit (Promega) based on TOPflash/FOPflash reporter plasmid system was used for the detection of β-catenin driven Wnt-transcriptional activity. The TOP Flash reporter construct contains three optimal copies of TCF/LEF sites upstream of a thymidine kinase minimal promoter that, when bound by β -catenin induces transcription of luciferase reporter gene. FOP Flash contains mutated copies of the TCF/LEF that cannot be activated by β -catenin and was used as negative control. Luciferase reporter gene analysis was performed using the SaOS cell line treated with either control unconditioned media or media conditioned by differentiating myogenic cells for 24 h. One day before transfection, 1 × 10^4^ cells/well were seeded per well into a 24-well plate. After overnight incubation, the cells (90% confluence) were transiently co-transfected with 0.4 mg DNA of reporter constructs (TOP/FOP Flash) and control plasmid (pCMV-GLuc) using 1 mL Lipofectamine 2000^TM^ (Invitrogen) in 50 mL OptiMEM I reduced serum media (Invitrogen, UK). Thereafter, the test cells were stimulated with control unconditioned media or conditioned media for 24 hours. Cells from the treated and control conditions were collected for dual luciferase assay (Firefly luciferase reporters and Renilla luciferase as internal control) according to manufacturer’s instructions. Each assay was performed in triplicate and the reporter activity expressed as mean ± SD.

### Lentiviral transduction

To generate lentiviral supernatants, HEK293T cells were cultured overnight in dishes pre-coated with 0.1 mg/ml collagen solution (Sigma, UK) in DMEM, 10% FCS, 2% glutamax and 1% pen/strep until 70% confluent. A solution containing 6.5 µg pCMV Δ8.9 packaging plasmid, 3.5 µg VSV-g envelope plasmid and 10 µg target construct were diluted in OptiMEM-1, combined with 30 µl Lipofectamine 2000 (LifeTechnologies, UK) and incubated at room temperature for 20 min to allow complexes to form. The plasmid/lipofectamine mixture was added dropwise to the HEK293 cells and incubated at 37 °C for 4 h before an equal volume of OptiMEM-1 was added. The lentiviral supernatant was collected 48 h post-transfection and concentrated using a lenti-X-concentrator kit (Clontech, USA) according to manufacturer’s specification and viral titre measured using GoStix (Clontech, USA). The pseudovirus was stored at −80 °C until ready to use. Fresh primary human muscle-derived cells at passage 1 were seeded in 6 or 24-well plates and maintained at 37 °C for 24 h in growth media prior to transduction. Human myogenic cells were transduced at a supernatant to media ratio ranging from 1:15 to 1:40 with 8 µg/ml polybrene (Sigma, UK) in growth media devoid of antibiotics. A mock control, consisting of the same transduction mixture without the addition of lentivirus was performed for each subject. The infected target cells were monitored for mCherry expression using fluorescent microscopy and after 48 h media was changed to growth media or differentiation media. In both cases the mouse ORF for β-catenin and TCF4/were expressed as β-catenin (central ARM domains) binding domains are 100% conserved between humans and mice. Primers were designed, which allowed us to measure endogenous transcripts to study activity at the host locus.

### Immunocytochemistry

Cryosections and cultured cells were fixed by the addition of 4% paraformaldehyde in ice-cold PBS for 10 minutes. Cells were permeabilised after fixation by addition of 0.2% Triton-X100 in PBS with 1% BSA and NaN_3_ (0.01%) for 10 minutes, then blocked and incubated with primary antibodies. This was followed by incubation with species-specific fluorescently-labelled secondary antibodies, and 10 minute incubation in Hoechst 33342 dye. Stained tissue sections and cells were mounted using ‘anti-fade gold’ medium (DAKO USA). Details of all antibodies used are shown in Supplementary Table 1.

### Image analysis

Immunofluorescent probes were illuminated by epifluorescence and signals were visualised through red (filter set 45 HQ Texas red shift free), green (filter set 44 FITC special shift free) and blue (filter set 49 DAPI shift free) band pass filters (Zeiss) on an AxioPlan microscope (Carl Zeiss; 10X, 20X, 40X, objectives with 0.25, 0.75, 0.95 numerical apertures respectively). Greyscale images were captured using an AxioCam MRm digital camera (Zeiss). To measure the expression of myogenic transcription factors and transcriptional co-activators in individual nuclei, image analysis was performed using techniques described in Agley *et al*.^6^. For all analysis 6–10 independent fields of view were analysed from two separate coverslips on cells derived from at least three representative myogenic cell populations. Only raw, greyscale images were used for quantitative analysis. Local background intensity values were subtracted from measurements to account for potential field to field variation in non-specific background fluorescence^62^.

### Western immunoblotting

Cell lysates were prepared using ice cold lysis buffer containing phosphatase and protease inhibitors (Roche, UK). Protein content of the samples was quantified using the Bradford assay (Bio-Rad, UK). Protein samples to be analysed were mixed at a 1:1 ratio with 2x Laemmli sample buffer and denaturing discontinuous SDS-PAGE was performed using 4% and 10% acrylamide: bis-acrylamide (37.5:1) stacking and resolving gels respectively. After SDS-PAGE had been performed, electro-blotting was performed using standard methods. Immobilised proteins were then probed overnight with primary antibodies. After washing, blots were incubated with the appropriate species-specific horseradish peroxidase (HRP)-conjugated secondary antibody (1:2000 dilution) for one hour at room temperature. Immuno-labelled protein bands were then visualised using ECL Plus Western Blotting Detection Reagents (Amersham Biosciences, UK) and ECL Hyperfilm (Amersham Biosciences, UK). Band density was quantified using Scion Image analysis software (Scion Image, release Alpha 4.0.3.2, USA). β-tubulin or GAPDH expression were used for the normalisation of protein loading. Details of all antibodies used are shown in Supplementary Table 1.

### Quantitative real-time polymerase chain reactions (qRT-PCR)

Cell monolayers were rinsed with PBS and cells lysed directly in their culture dishes by the addition of TRIzol reagent (Invitrogen, UK). Isolation of RNA from TRIzol was performed as per the manufacturer’s instructions. RNA was reverse transcribed using the QuantiTect Reverse Transcription kit (Qiagen, UK) and RotorGene 6000 (Qiagen, UK) qPCR machine following the manufacturer’s instructions. qPCR was performed as described by Bailey *et al*.^63^ and normalised to 4 stable reference genes or as performed by Lewis *et al*.^64^. Primer sequences can be found in Supplementary Table 2.

### Statistics

For experiments comparing only two groups, data were compared by unpaired t-test with Welch’s correction, which does not assume equal variances between groups; where data did not satisfy all parametric assumptions Mann-Whitney U tests were used to compare differences between groups. For all experiments comparing more than two groups a one or two way (if repeated measures or time as a factor) analysis of variance (ANOVA) was performed and specific time points were analysed via post-hoc Bonferroni’s multiple comparison test. In all cases *n* numbers are given for specific experiments and an alpha value of *P* < 0.05 was accepted as statistically significant. For quantification of immunofluorescent staining 6–10 independent fields of view were analysed per condition (pooled from two separate coverslips) on cells derived from at least 2–3 representative myogenic populations per experiment. In all other cases *n* numbers are given for specific experiments and an alpha value of *P* < 0.05 was accepted as statistically significant.

## Electronic supplementary material

Supplementary Information
