# Supplementary material for: Active GSK3β and an intact β-catenin TCF complex are essential for the differentiation of human myogenic progenitor cells
Source: Sci Rep. 2017 Oct 13;7:13189. doi: 10.1038/s41598-017-10731-1 (PMC5640663; doi:10.1038/s41598-017-10731-1)
Supplement: Supplementary file 1 — Supplementary Information [file 41598_2017_10731_MOESM1_ESM.pdf]

Active GSK3β and an intact β-catenin TCF complex are essential for the differentiation of human myogenic progenitor cells

Agley C.C.*† [1, 4], Lewis, F.C. † [1,2], Jaka O.[1], Lazarus N.R.[1], Velloso, C.[1], Francis-West P.[3], Ellison-Hughes, G.M.[1,2] and Harridge S.D.R.[1,2]

Supplementary Figure S1

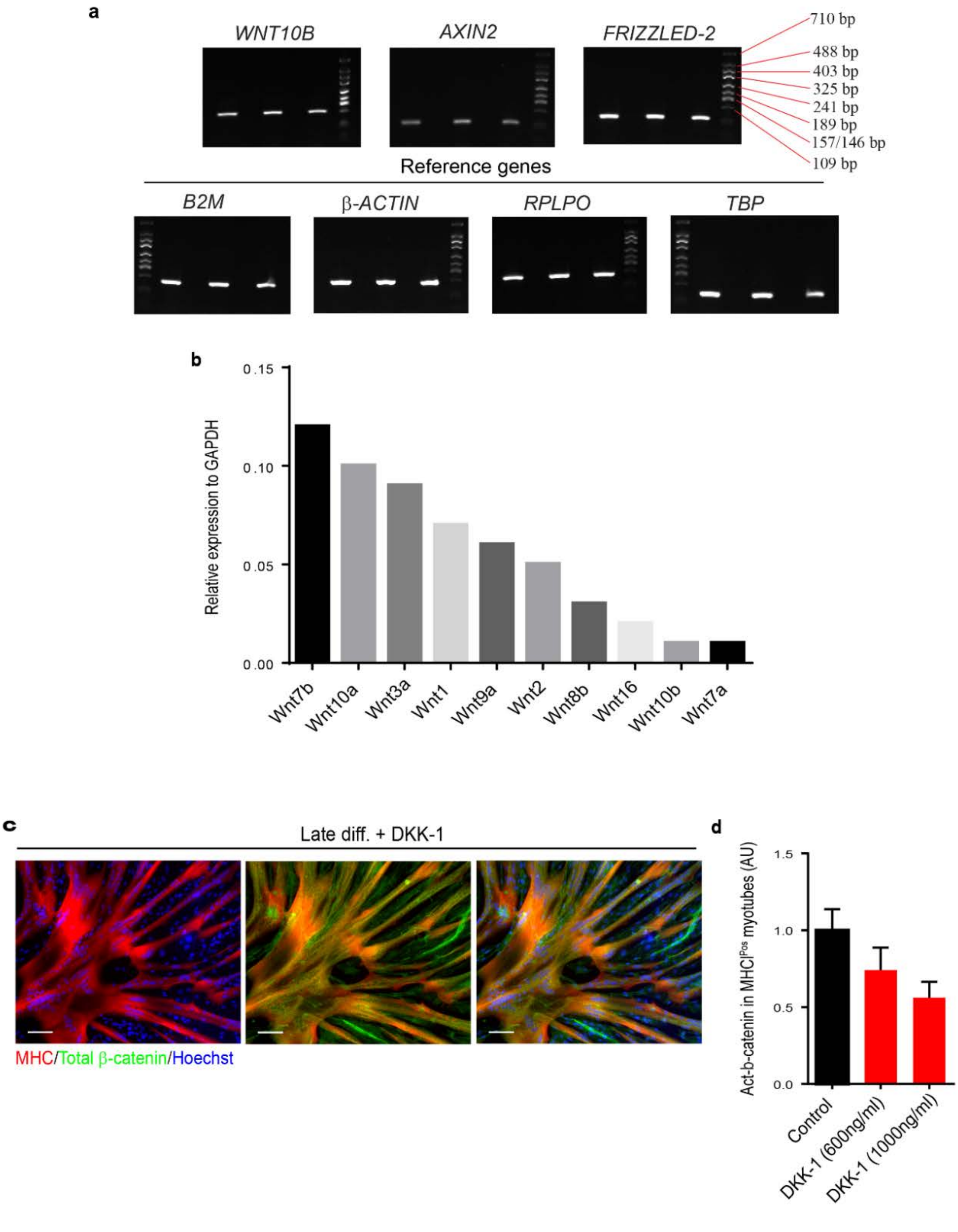

Figure S1. ***Wnt signalling components in human myotube formation*** (**a**) PCR analysis of Wnt signalling components in human muscle-derived myogenic cell populations from healthy individuals. (**b**) qRT-PCR analysis of human Wnts expression in differentiating myogenic cells (2-3d). (**c**) Representative image of human myogenic cells differentiated for four days in the presence of Dkk-1 (300ng/ml) shows the formation of large MHC^{pos} myotubes and abundant expression of total-β-catenin, scale bar=100µm. (**d**) Immunofluorescent quantification of active-β-catenin in MHC^{pos} human myogenic cells differentiated for four days in the presence of different doses of Dkk-1 (600ng/ml and 1000ng/ml). All bar charts are means ± SD. For quantitation of immunofluorescence 6-10 independent fields of view were analysed per condition from *n* = 3 representative myogenic cell populations.

Supplementary Figure S2

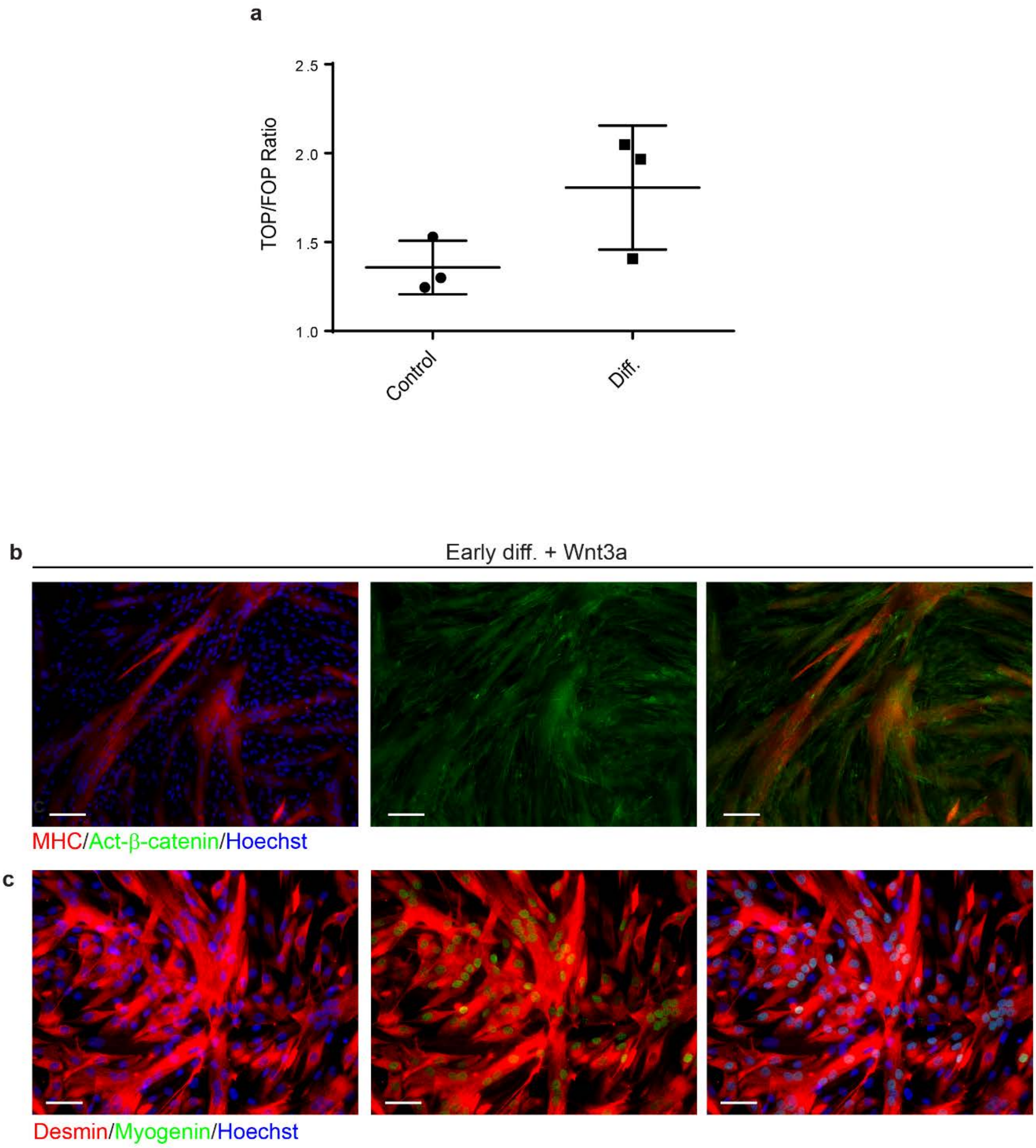

Figure S2. ***Induction of β-catenin and MRFs following myogenic cell fusion and early differentiation*** (**a**) The TCF transcriptional activity of SAOS cells is shown as a ratio of TOP-FLASH to FOP-FLASH luciferase-mediated signals, when cultured in the presence of conditioned media from differentiating myogenic cells (day 2-3). Each assay was performed in triplicate and the reporter activity expressed as mean ±SD, *n* = 3 representative myogenic cell populations. (**b**) Purified myogenic cells cultured in differentiation medium supplemented with Wnt3a (400ng/ml) (24h) express act-β-catenin and moderate levels of MHC. (**c**) Desmin^{Pos} myogenic cells upregulate myogenin when switched to differentiation media supplemented with Wnt3a (400ng/ml) (24h), scale bars=100µm top panel, 50µm bottom panel.

Supplemental Figure S3

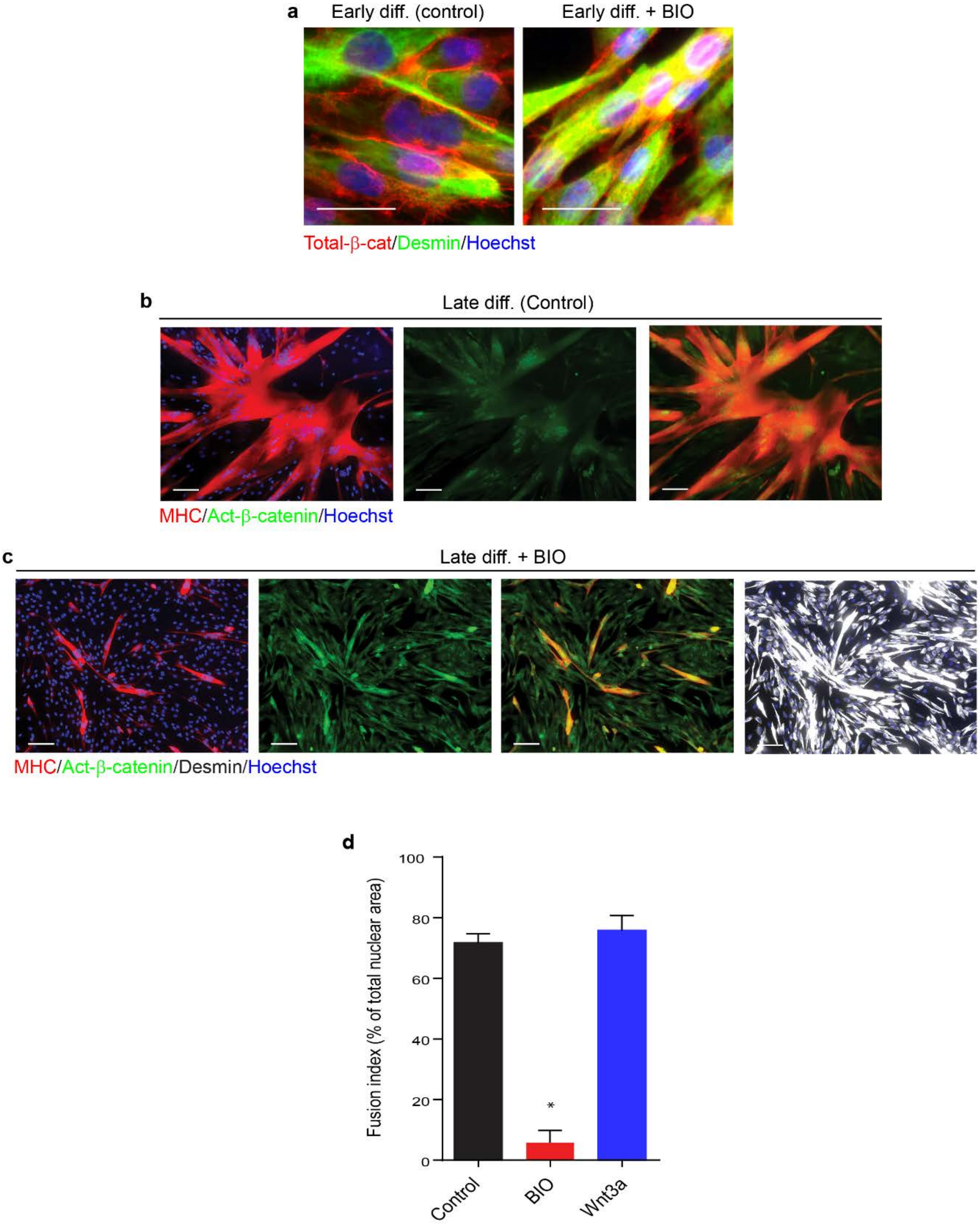

Figure S3. *β-catenin and MHC expression in BIO-treated myogenic cultures* (**a**) Representative micrograph shows Total-β-catenin immunostaining of myogenic cell populations after 24 hours in differentiation media with and without BIO, scale bar=10μm (**b**) Representative micrograph shows MHC/Active-β-catenin immunostaining of myogenic cell populations after 4 days in differentiation media. (**b**) Representative micrograph shows MHC/Active-β-catenin/Desmin immunostaining of myogenic cultures treated with BIO for 4 days in differentiation media. All scale bars=100μm. (**c**) Quantification of myotube fusion index in control, BIO and Wnt3a-treated myogenic cells. All bar charts are means ± SD. * *P* <0.05 compared to both control and Wnt3a-treated human myogenic cultures.

Supplemental Figure S4

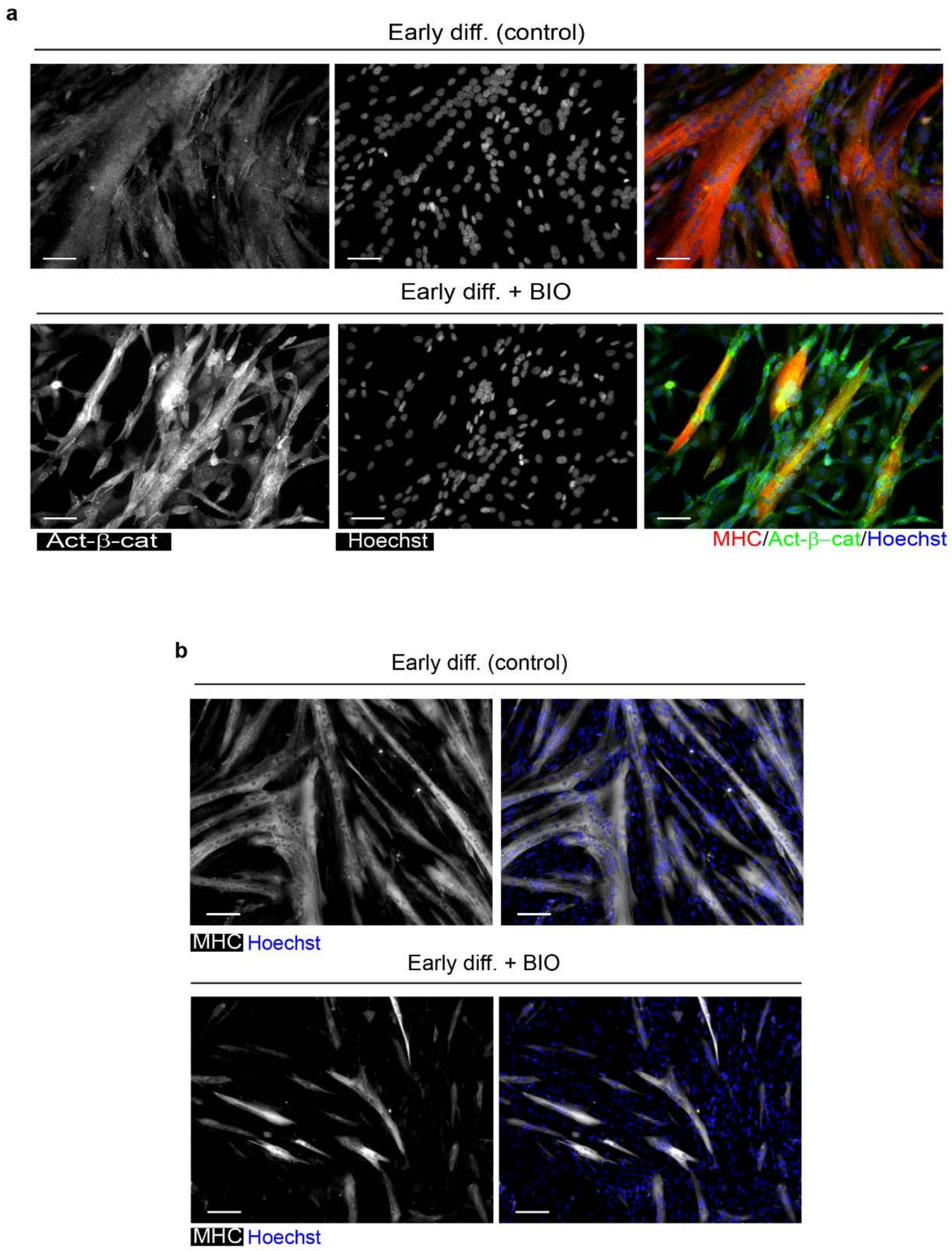

Figure S4. ***Greyscale micrographs illustrate cellular changes occurring in early differentiation in BIO-treated myogenic cultures*** (**a**) Representative grey scale micrograph shows increased nuclear Act-β-catenin immunostaining of myogenic cell populations after 24 hours BIO-treatment. (**b**) Representative grey scale micrograph shows increased number of small MHC^{pos} myotubes after 24 hours BIO-treatment. All scale bars=100µm

Supplemental Figure S5

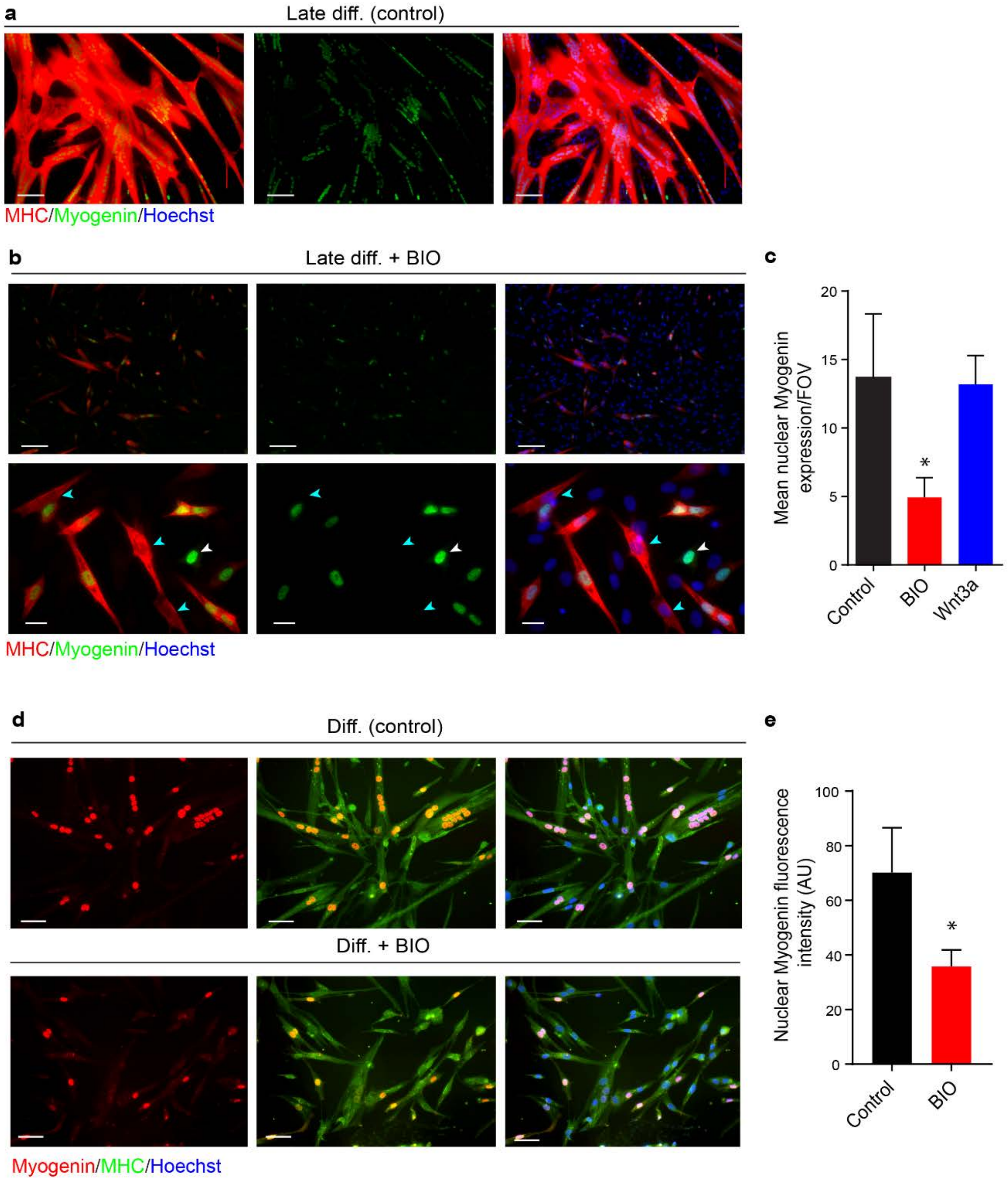

Figure S5. *Myogenin expression in BIO-treated myogenic cultures* (**a**) Representative micrograph shows MHC/myogenin immunostaining of purified myogenic cells cultured in differentiation medium for 4 days. (**b**) Representative micrograph shows MHC/myogenin immunostaining of myogenic cells cultured in differentiation medium for 4 days in the presence of BIO. Cell by cell analysis reveals an increase in the abundance of myogenin^{Pos}/MHC^{Neg} (white arrows) and to a greater extent MHC^{Pos}/myogenin^{Neg} (blue arrows) in BIO cultures. (**c**) Quantification of mean nuclear myogenin expression per field of view. (**d**) Representative micrograph showing myogenic cells that were differentiated for 2 days to permit myogenin expression then BIO applied for a further 24h, which is shown to inhibit myogenin expression. (**e**) Quantification of mean nuclear myogenin fluorescence intensity. All scale bars=100µm, except (**b**) bottom panel =20µm. Bar charts are means ± SD. * *P* <0.05 compared to control and Wnt3a-treated human myogenic cultures.

Supplementary Figure S6

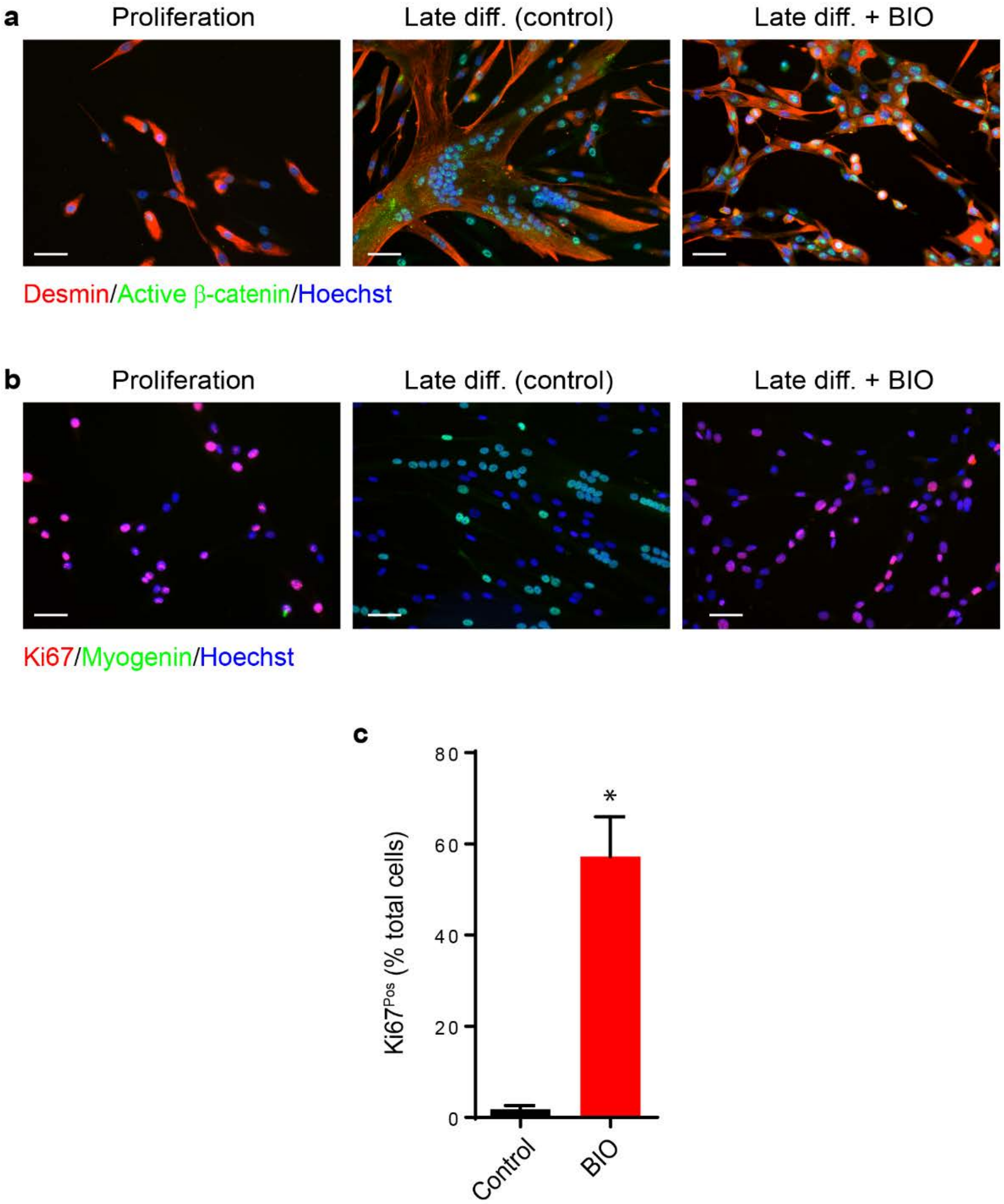

Figure S6. ***Ki67 expression in BIO-treated myogenic cultures*** (**a**) Representative micrograph shows desmin/active β-catenin immunostaining of purified myogenic cells cultured in either proliferation (1d), differentiation (3d) or differentiation media + BIO (3d). (**b**) Representative micrograph shows Ki67/myogenin immunostaining of myogenic cells cultured under the same conditions, scale bars=100µm. (**c**) Quantification of Ki67^{Pos} cells per total number of cells. Bar chart represents mean ± SD. * *P* <0.05 compared to either control cultures.

Supplementary Figure S7

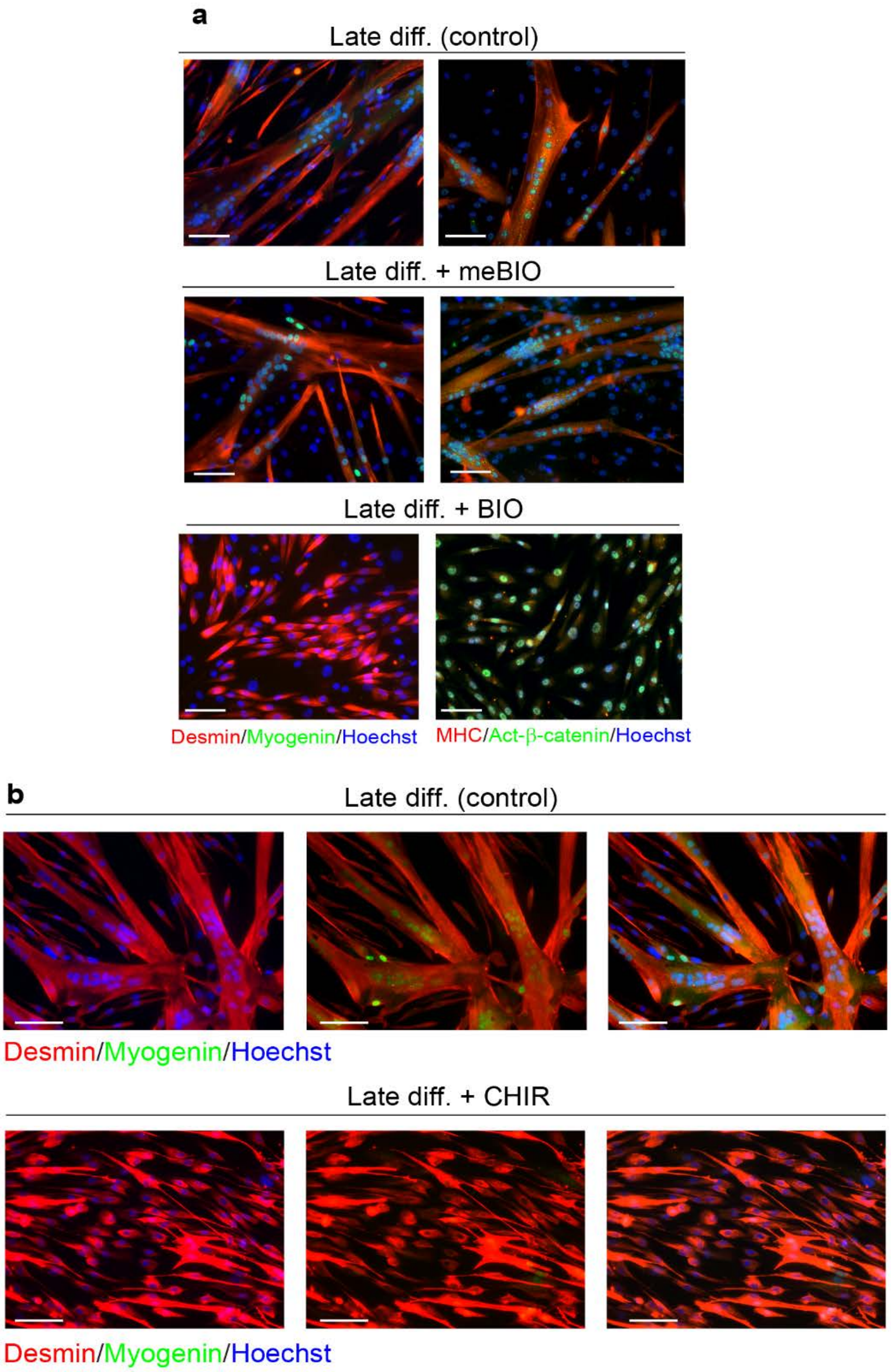

Figure S7. ***Effect of BIO on active-β-catenin and myosin heavy chain expression*** (**a**) Representative micrograph of desmin/myogenin or MHC/active-β-catenin immunostaining of human myogenic cells exposed to control differentiation conditions, meBIO or BIO (4 days). (**b**) Representative micrograph of desmin/myogenin immunostaining of human myogenic cells exposed to control differentiation conditions or CHIR (4 days), scale bars=100µm.

Supplementary Figure S8

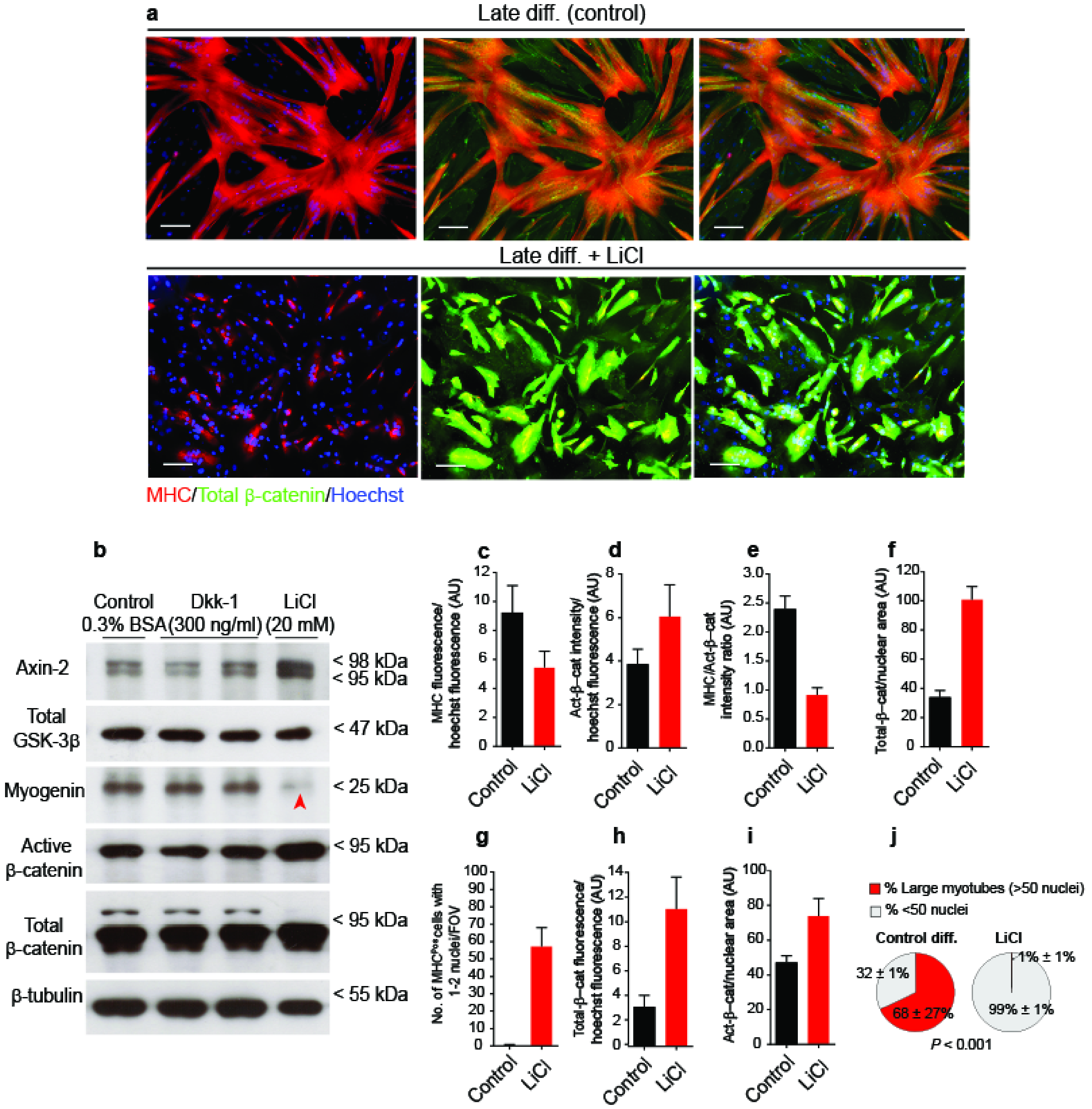

Figure S8. ***Effect of LiCl on β-catenin expression and myogenic cell differentiation*** (**a**) Representative image of total β-catenin and MHC expression in human myogenic cells differentiated for 4 days +/- LiCl (20mM), scale bars=100µm. (**b**) Western immunoblotting of human myogenic cells subjected to differentiation in the presence of Dkk-1 (300ng/ml) or LiCl (20mM) show that Dkk-1 had no observable effect on any of the proteins studied while LiCl increased expression of Axin2, active-β-catenin and total-β-catenin. Myogenin expression was also severely blunted in LiCl treated cells (red arrowhead). (**c**) Fluorescence intensity of MHC in control and LiCl-treated cultures (4 days). (**d**) Fluorescence intensity of act-β-catenin in control and LiCl-treated cultures (4d). (**e**) The ratio of MHC fluorescence to act-β-cat fluorescence in LiCl-treated cultures compared to control myogenic cultures (4 days). (**f**) Total β-catenin/nuclear area in control and myogenic cultures exposed to LiCl (4 days). (**g**) Quantification of MHC^{pos} myotubes containing only 1-2 nuclei in control and LiCl-treated cultures (4 days). (**h**) Fluorescence intensity of total-β-catenin/hoechst in cultures supplemented with LiCl compared to control. (**i**) Fluorescence intensity of active-β-catenin/hoechst in cultures supplemented with LiCl compared to control. (**j**) Quantification of myogenic fusion index in control and LiCl-treated cultures. All bar charts are means ± SD.

Supplementary Figure S9

a

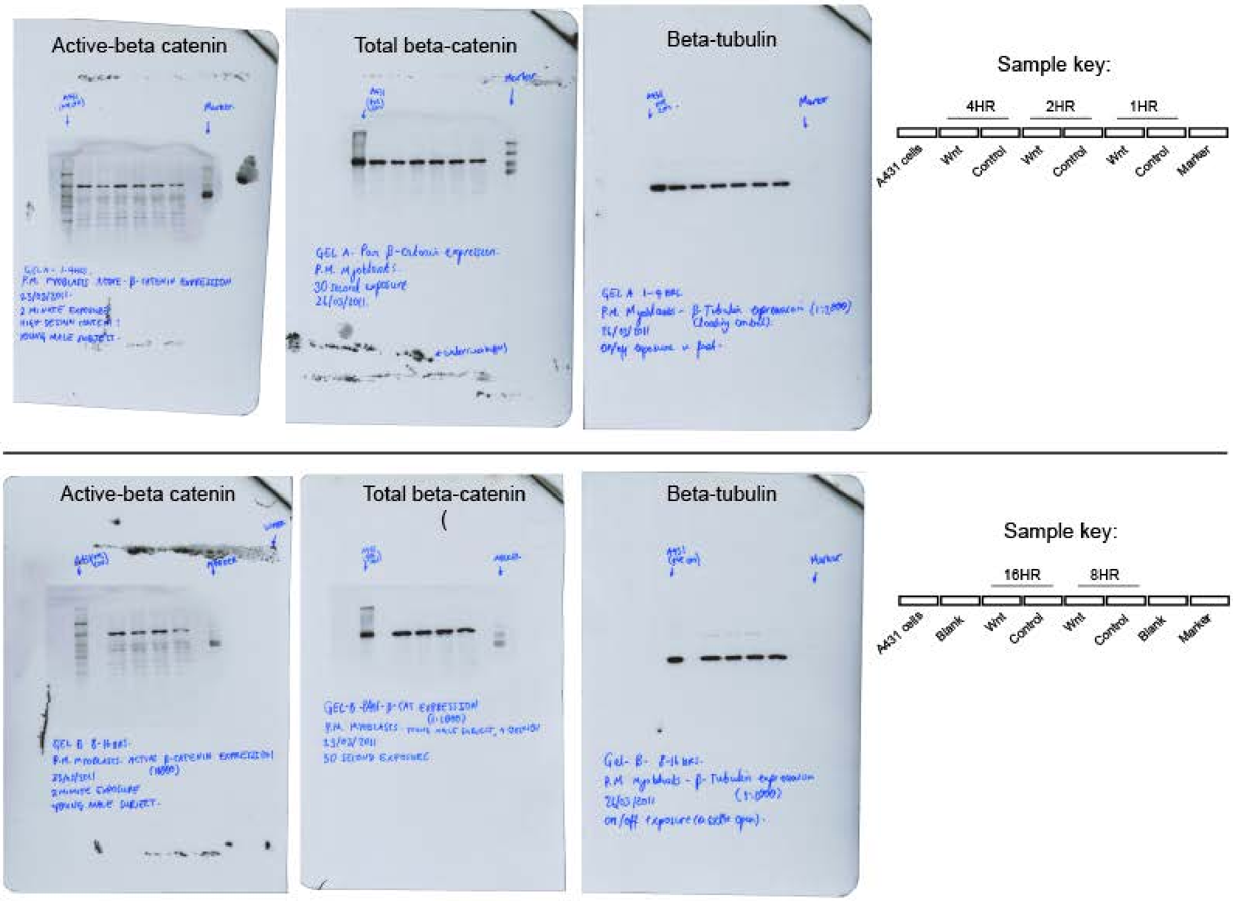

b

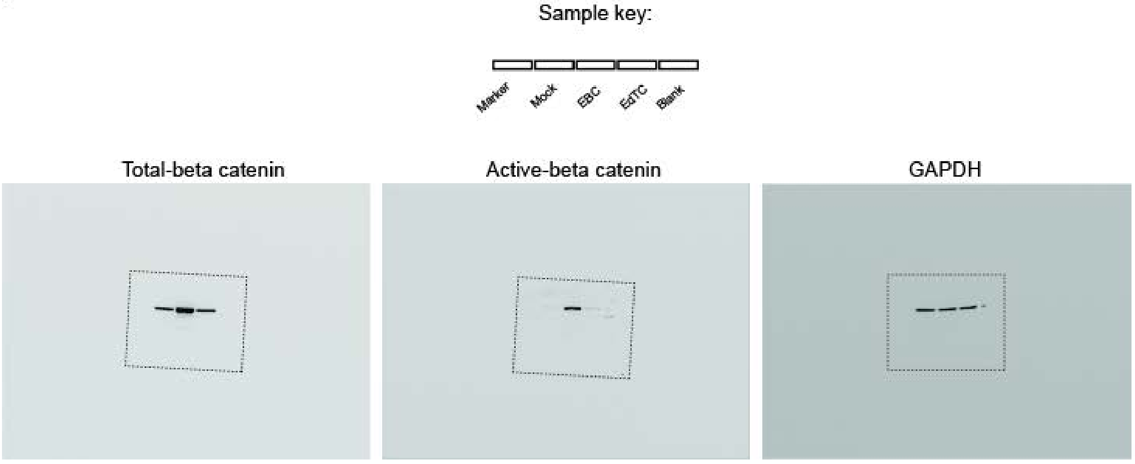

Figure S9. Original western blot gels (**a**) Individual western blot gels associated with figure 2a. (**b**) Individual western blot gels associated with figure 5c.

Table 1

Antigen	Speciation	Isotype	Clone	Clonality	Dilution	Supplier
**CD56**	Mouse	IgG1	MY-31	Monoclonal	1/100	BD
**Laminin**	Rabbit	IgG	N/A	Polyclonal	1/250	Dako
**Act-β-catenin**	Mouse	IgG1κ	8E 7	Monoclonal	1/150	Millipore
**Total β-catenin**	Mouse	IgG1	L87A12	Monoclonal	1/150	NEB
**Total β-catenin**	Rabbit	IgG	D10A8	Monoclonal	1/150	NEB
**Desmin**	Rabbit	IgG1	D93F5	Monoclonal	1/250	NEB
**Desmin**	Mouse	IgG1	D33	Monoclonal	1/250	Dako
**Ki67**	Rabbit	IgG	SP6	Monoclonal	1/200	MD
**MHC**	Mouse	IgG2b	MF20	Monoclonal	1/200	DSHB
**Myogenin**	Mouse	IgG1	F5D	Monoclonal	1/50	DSHB
**GSK-3β (total)**	Mouse	IgG1	27C10	Monoclonal	1/500	NEB
**Axin-2**	Rabbit	IgG	D48G4	Monoclonal	1/150	NEB
**β-tubulin**	Mouse	IgG1	E7	Monoclonal	1/1000	DSHB

Table 1. Primary antibodies used in immunocytochemical analysis.

Table 2

Gene	Forward Primer	Reverse Primer	Tm	Accession
**B2M**	TTCTGGCCTGGAGGCTATC	TCAGGAAATTTGACTTTCCATTC	60	NM_004048.2
**β-ACTIN**	GTGGCATCCACGAAACTACC	GTACTTGCGCTCAGGAGGAG	60	NM_001101.3
**RPLP0**	TCTACAACCCTGAAGTGCTTGAT	CAATCTGCAGACAGACACTGG	60	NM_001002.3
**TBP**	CGGCTGTTTAACTTCGCTTC	CACACGCCAAGAAACAGTGA	60	NM_003194.4
**GAPDH**	TGCACCACCAACTGCTTAGC	GGCATGGACTGTGGTCATGAG	60	NM_002046
**β-CATENIN**	TTGTGCGGCGCCATTTTAAG	ATTAACCACCACCTGGTCCTC	60	NM_001904.3
**TCF4**	CCATCTCTCTCAGCAGGCAC	GGTGTCAGGTCCTCATCGTC	60	NM_001083962.1
**AXIN-2**	CCACACCCTTCTCCAATCC	TGCCAGTTTCTTTGGCTCTT	60	NM_004655.3
**MYF5**	TACCGGAGCGACAGACTAGG	GTTACATTCGGGCATGCCATC	60	NM_005593.2
**MYOD**	ACGGCATGATGGACTACAGC	TGGGTTACGGTTACACCTGC	60	NM_002478.4
**MYOG**	CCCTGAAGAGAAGCACCCTG	CAGATGATCCCCTGGGTTGG	60	NM_002479.5
**MYF5**	TGGAAATCAGTTATAGGGAGTTTT	TTTGTGCTTACATTAAAAAGATGC	60	Y17154
**MYOD**	TGCGTATTCTCAACCCCTTC	AGTATGCAAGGGTGGAGTGG	60	U12574
**MYOGENIN**	GAAGGTGAATGAGGCCTTTG	TGTGGGAACTGCATTCACTG	60	NM_001012406.1
**MHC**	CTACGCCAGGGTCCTTAACTG	TCCAGAGTCCCCTGCATTTTG	60	NM_005963.3
**FRIZZLED-2**	GGTGTCGGTGGCCTACAT	GAGAAGCGCTCGTTGCAC	60	NM_001466.2
**WNT1**	GCCGTACGACCGTATTCTCC	TGCTAGCGAGTCTGTTTGGG	60	NM_005430.3
**WNT2**	GCTGGAATTGCAACACCCTG	ACCGCTTTACAGCCTTCCTG	60	NM_003391.2
**WNT3A**	GGACAAAGCTACCAGGGAGTC	AGAGGAGACACTAGCTCCAGG	60	NM_033131.3
**WNT7A**	GGGACTATGAACCGGAAAGCG	ATGTTCTCCTCCAGGATCTTTCG	60	NM_004625.3
**WNT7B**	GTACGTGAAGCTCGGAGCAC	ACTGGTACTGGCACTCGTTG	60	NM_058238.2
**WNT8B**	TGACTGGTCCAAAGGCTTACC	GTTTTCTCCCGGGTTTGTGC	60	NM_003393.3
**WNT9A**	TCGAGTGCCAGTTCCAGTTC	TGATCACCTTCACACCCACG	60	NM_003395.2
**WNT10B**	GCGAATCCACAACAACAGG	TCCAGCATGTCTTGAACTGG	60	NM_003394.2
**WNT16**	TACAGCTCCCTGCAAACGAG	CAATGCCCAACCACATCCAG	60	NM_057168.1

Table 2. PCR primers used in qRT-PCR reactions.
